# Identification and verification of ferroptosis-related core gene in postmenopausal osteoporosis based on bioinformatics analysis

**DOI:** 10.7717/peerj.20666

**Published:** 2026-03-31

**Authors:** Dongcheng Ran, Cheng Yang, Enyu Zhou, Weimin Li, Jiamu Xu, Wangxiang Wu, Chunqing Wang

**Affiliations:** 1 Department of Orthopedic, Beijing Jishuitan Hospital Guizhou Hospital, Guiyang, Guizhou Province, China; 2 Tumor Research Laboratory, The Affiliated Cancer Hospital of Guizhou Medical University, Guiyang, China; 3 Emergency Department, The Affiliated Hospital of Guizhou Medical University, Guiyang, China

**Keywords:** Postmenopausal osteoporosis, Bioinformatics, Ferroptosis, Core gene

## Abstract

**Objective and Background:**

Postmenopausal osteoporosis (PMOP) significantly increases the risk of fragility fractures and has substantial negative effects on patients. Accumulating evidence suggests that the pathogenesis of PMOP is associated with ferroptosis. In this study, we screened and validated core ferroptosis-related genes (FRGs) in PMOP and investigated their potential links using bioinformatics analysis.

**Methods:**

Differentially expressed genes (DEGs) were identified between PMOP patients and healthy postmenopausal women based on the GSE230665 dataset in the Gene Expression Omnibus (GEO) database. Weighted gene co-expression network analysis (WGCNA) was conducted to sort and extract significant module genes. FRGs were acquired from an online ferroptosis database. By intersecting DEGs, FRGs, and significant module genes, we identified ferroptosis-associated DEGs (FDEGs). We performed Gene Ontology (GO), Kyoto Encyclopedia of Genes and Genomes (KEGG), and protein-protein interaction (PPI) analyses on FDEGs. Multiple algorithms within the cytoHubba plugin were employed to detect the core gene from the PPI network. The expression of the core gene was subsequently validated in the PMOP rat model, and the diagnostic efficacy of the core gene was evaluated using receiver operating characteristic (ROC) curves applied to external datasets. GeneMANIA and Gene Set Enrichment Analysis (GSEA) were conducted to explore important functions and pathways of the core gene. We further utilized the CIBERSORTx website to evaluate immune infiltration in PMOP and molecular docking analysis was performed to develop potential drugs for PMOP. Finally, we established a transcription factors (TFs)-mRNA-miRNAs regulatory network for mechanisms investigation.

**Results:**

In this study, we identified phosphatase and tensin homologue deleted on chromosome 10 (PTEN) as the core gene through PPI analysis and various algorithms. qRT-PCR analysis and immunohistochemical staining revealed a significant elevation of PTEN expression in the PMOP rat models, and ROC curves demonstrated the relatively high diagnostic properties of PTEN. Moreover, GSEA analysis indicated that PTEN is involved in functions and pathways associated with MAPK signaling, oxidative phosphorylation, mTOR signaling, *etc.* We discovered three drugs that bind tightly to PTEN, including CX-5461, UMI-77, and GSK2256098. Ultimately, we constructed a TFs-mRNA-miRNAs network comprising 39 nodes (16 TFs, 22 miRNAs, and 1 mRNA) and 38 edges.

**Conclusion:**

PTEN was preliminarily identified and verified as a key ferroptosis gene in postmenopausal osteoporosis through bioinformatics analysis, and the diagnostic model constructed based on PTEN was of high value. These findings may provide novel perspectives on the pathogenesis of PMOP at the transcriptome level and establish a theoretical foundation for further research.

## Introduction

The dynamic balance between osteoblast-driven bone formation and osteoclast-mediated bone absorption is needed to maintain bone homeostasis, and the role of estrogen in promoting osteoblast maturation and inducing osteoclast apoptosis is critical for preserving this balance (Cline-Smith et al., 2020; Kim & Koh, 2019). Postmenopausal women are more vulnerable to osteoporosis (OP) because bone resorption exceeds bone formation during menopause, owing to reduced ovarian function and decreased estrogen levels (Awasthi et al., 2018). Postmenopausal osteoporosis (PMOP) commonly affects women within 5–10 years of menopause and is distinguished by diminished bone mass and deteriorating bone microstructure that increases bone fragility and fracture risk (Black & Rosen, 2016; Zhao et al., 2018). The prevalence of PMOP is rapidly increasing worldwide as the global population progressively ages. Moreover, about 50% of postmenopausal women globally develop OP, and 40% of them experience fractures (Rachner, Khosla & Hofbauer, 2011). The most frequent and dangerous side effects of PMOP are fragility fractures that frequently occur in the hip, spine, or femur under non-traumatic or slightly stressful circumstances; these result not only in pain, deformity, and dysfunction but also in death (Xu et al., 2017). The World Health Organization has ranked PMOP-associated fractures as the third greatest threat to the life and health of older populations because of the increased incidence of disability and mortality, high economic costs, and tremendous social burden (Guo et al., 2021; Brenneman et al., 2006; Reid, 2020). However, the pathogenesis of PMOP remains obscure. Therefore, a thorough investigation of the biomarkers or targets involved in PMOP pathogenesis is essential to developing novel preventive and treatment strategies.

Ferroptosis, a novel programmed cell death, is different from necrosis (Zou et al., 2021), autophagy (Yoshida et al., 2022), pyroptosis (Tao et al., 2020), and necroptosis (Galluzzi et al., 2017), and it participates in the processes of many pathological states. It is typically manifested by intracellular iron and iron ion accumulation through the Fenton reaction. Abundant reactive oxygen species (ROS) that disrupt the cellular antioxidant/pro-oxidant homeostatic system consequently damage nucleic acids, proteins, and lipids (Zhao, 2019; Dixon et al., 2012). Smaller and rounded cells, intercellular separation, intact and shrunken cell membranes, blistered plasma membranes, wrinkled mitochondria, and ruptured mitochondrial membranes are the major morphological characteristics of cellular ferroptosis (Kazan & Kalaipandian, 2019). Osteoarthritis, tumors, cardiovascular diseases, ischemic injury, and kidney damage are associated with ferroptosis (Fang et al., 2023; Wang et al., 2022; Jiang, Stockwell & Conrad, 2021). Postmenopausal women can develop signs of iron overload in addition to estrogen deficiency, and iron ion chelators can inhibit osteoclast differentiation and iron accumulation and decrease bone resorption (Chen et al., 2015). Moreover, bone marrow-derived macrophages that are stimulated to generate osteoclasts respond favorably to iron ions and are associated with increased ROS levels and oxidative stress (Jia et al., 2012). Thus, further investigation of the underlying molecular regulatory mechanisms is crucial, as ferroptosis might be important in PMOP development.

**Figure 1:**
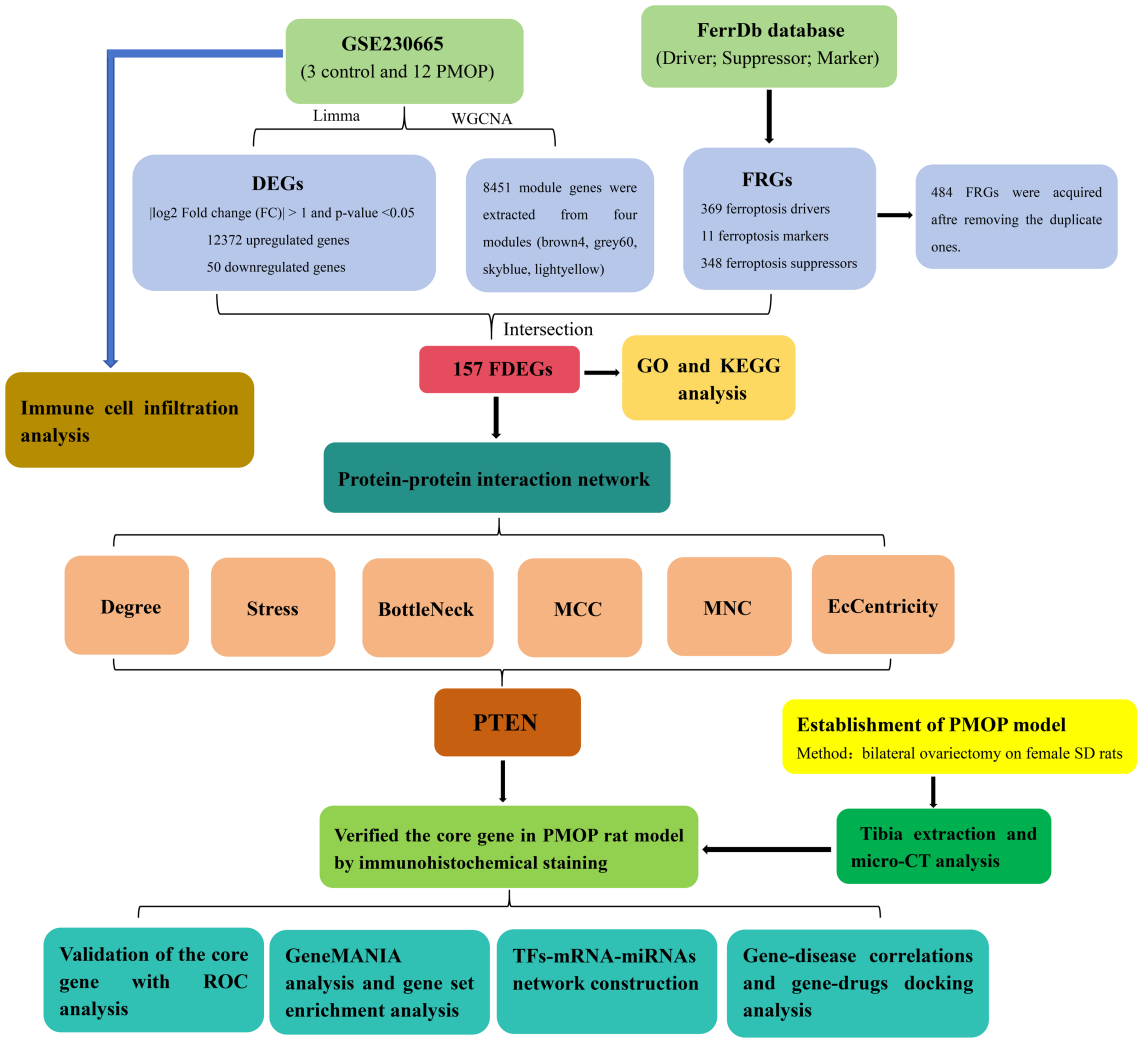
Flowchart of this study.

Bioinformatic analysis can identify innovative targets for PMOP progression and prognosis and aid in understanding the processes underlying PMOP incidence and development (Song et al., 2020). At present, there are very few bioinformatics studies on ferroptosis in PMOP, and identifying ferroptosis-related genes (FRGs) as possible biomarkers of PMOP is of great research significance. Here, we determined a core gene with extremely robust connectedness that was prominently positioned in protein-protein interaction (PPI) networks and might serve as a target for diagnosing disease based on bioinformatics analysis. We then verified the core gene’s expression by establishing rat models of PMOP and discovered significant differences. Therefore, this study has a certain degree of novelty and provides a theoretical basis for subsequent research related to ferroptosis in PMOP. A flowchart of this study is shown in Fig. 1.

## Material and Methods

### Data acquisition

The microarray data of PMOP and healthy postmenopausal femoral tissues were acquired from the Gene Expression Omnibus (GEO; https://www.ncbi.nlm.nih.gov/geo) database (Patra et al., 2020). We selected the GSE230665 dataset, and the platform information was GPL10332 (Agilent-026652 Whole Human Genome Microarray 4x44K v2). The array data of GSE230665 consisted of twelve samples of postmenopausal osteoporosis patients (GSM7230907, GSM7230908, GSM7230909, GSM7230910, GSM7230911, GSM7230912, GSM7230913, GSM7230914, GSM7230915, GSM7230916, GSM7230917, and GSM7230918; PMOP group) and three healthy postmenopausal women (GSM7230904, GSM7230905, GSM7230906; control group). All data were freely accessible, and the detailed data of patients was in Table S1. Meanwhile, two datasets, GSE7429 (10 control samples and 10 PMOP samples) and GSE56116 (three control samples and three PMOP samples), were downloaded as external datasets.

### Identification of differentially expressed genes (DEGs)

R (version 4.4.1; R Core Team, 2024) was applied to transform the probe name into gene symbol. When several probes matched one gene, the average expression value was calculated. Furthermore, *p*-value <0.05 and —log2 Fold change (FC)—>1 were set as the criterion to detect DEGs using the limma package (version 3.40.6) (Ritchie et al., 2015). The ferroptosis database (FerrDb; http://www.zhounan.org/ferrdb) provided 484 FRGs, including ferroptosis suppressor, ferroptosis driver, and ferroptosis marker genes (Zhou & Bao, 2020).

### Weighted gene co-expression network analysis

In this study, we utilized the WGCNA package (version 1.73) to perform gene correlation analysis (Langfelder & Horvath, 2008). Initially, we calculated the MAD (Median Absolute Deviation) of every gene and excluded the bottom 50% of genes with the lowest MAD values. Subsequently, we constructed a scale-free co-expression network by removing outlier genes and samples utilizing the goodSamplesGenes method. Following this, Pearson’s correlation analysis was conducted for all genes to generate a weighted adjacency matrix. The soft-thresholding exponent, *β*, could effectively accentuate robust gene correlations while diminishing the influence of weaker associations. After applying an exponent of 9, the adjacency matrix was transformed into a topological overlap matrix (TOM). In the next step, we employed average linkage hierarchical clustering based on the TOM-derived dissimilarity measure to group genes with similar expression profiles into modules, setting a minimum module size of 30 for the gene dendrogram. To further analyze the identified modules, we calculated the dissimilarity of module eigengenes, established an appropriate cut-off point for the module dendrogram, and merged certain modules. Finally, the Pearson correlation coefficient was used to analyze the correlation between gene modules and PMOP. The top four gene modules closely related to PMOP were defined as key modules, and the significant module genes were extracted for further study. Through the intersection of DEGs, FRGs, and module genes, the PMOP-related ferroptosis DEGs (FDEGs) were obtained.

### Functional enrichment analysis and visualization

Gene ontology (GO) categorizes and annotates genes according to biological processes (BP), cellular components (CC), and molecular functions (MF) (Gaudet et al., 2021). Kyoto Encyclopedia of Genes and Genomes (KEGG) pathway enrichment is a vital analytical method for investigating gene functions at the level of molecular signaling networks (Kanehisa et al., 2023). GO and KEGG analyses were performed on the FDEGs through the R software package clusterProfiler with the criterion of *P* < 0.05 (version 3.14.3) (Yu et al., 2012), and the results were presented *via* the sangerbox online platform (http://vip.sangerbox.com/).

### Construction of the protein-protein interaction (PPI) network and identification of gene clusters

We created and assessed the PPI network with the help of the STRING database (version 11.5; https://cn.string-db.org/) (Szklarczyk et al., 2019). Cytoscape software (version 3.9.1; https://cytoscape.org) enables topological investigation of PPI networks and displays data outcomes (Shannon et al., 2003). The FDEGs were inserted into the STRING database to explore their interconnections. A PPI file downloaded from STRING was submitted to Cytoscape to generate a network graph with nodes indicating genes and edges reflecting connections among them. Then, the tightly interacting cluster modules were determined using Molecular Complex Detection (MCODE; version 2.0.2).

### Identification and validation of core gene

We screened the top five genes from the PPI network using six algorithms respectively *via* the cytoHubba plugin (version 0.1) in the Cytoscape software, namely Degree, Stress, BottleNeck, MCC, EcCentricity, and MNC (Chin et al., 2014). Then these genes were taken to intersect to generate the core gene of ferroptosis in PMOP.

### Animals

We acquired six female Sprague-Dawley (SD) rats from the animal research center at Guizhou Medical University, and the Animal Care Welfare Committee of Guizhou Medical University approved all the animal experiments in this study (approval number: 2305164).

### Establishment of PMOP model rats

Six healthy 6-month-old female SD rats (weight 300–350 g) were completely randomly allocated to experimental group (ovariectomized, OVX) or control group (sham-operated) (*n* = 3 per group) by using random control table. The rats underwent anesthesia with an intraperitoneal injection of 1% pentobarbital (0.4−0.5 mL/100 g). The abdominal cavities of the rats were opened with a longitudinal incision of three centimeters down the middle of the back. Pinkish ovaries were located along white adipose tissues using arterial forceps, then the fallopian tubes and ovarian arteries were tied with sutures, and bilateral ovaries were removed. A small portion of fatty tissue surrounding the ovaries was excised from the sham group. After the operation, all rats were kept in captivity under standard laboratory conditions (alternating 12 h of light and 12 h of darkness, temperature 25 ± 2 °C, humidity 55 ± 5%) with water and appropriate rodent food. Postoperative wound healing, diet, and daily activities were monitored. All rats gradually recovered and moved freely, with normal wound healing.

### Tibia extraction and micro-computed tomography (micro-CT) analysis

All rats were euthanized by intraperitoneal injection with a pentobarbital overdose on postoperative week 12. The bilateral tibias were excised, fixed for about 48 h in 4% paraformaldehyde, and immersed in 75% alcohol. Then, the tibias (*n* = 6 per group) were scanned at a resolution of 12 µm using a Skyscan 1275 high-resolution micro-CT system (Bruker Belgium SA, Kontich, Belgium). Thereafter, three-dimensional histomorphometric images of the tibias were reconstructed using NRecon software (Bruker Belgium SA). To assess the bone mass to determine whether the PMOP had been established, we gathered the following parameters: bone volume (BV), trabecular bone volume per tissue volume (BV/TV), trabecular separation (Tb. Sp), and trabecular number (Tb. N).

### Real-time quantitative reverse transcription polymerase chain reaction

Total RNA was extracted from the left femoral tissues of two groups applying TRIzol (Invitrogen) reagent and the RNA concentration was measured using the NanoDrop 2000 (Thermo Fisher Scientific). cDNA was transcribed by employing SweScript All-in-One SuperMix for qualitative polymerase chain reaction (qPCR) kit (Wuhan Servicebio Technology Co. Ltd., Wuhan, China), and real-time quantitative reverse transcription polymerase chain reaction (qRT-PCR) was performed using SYBR Green qPCR Master Mix (Wuhan Servicebio Technology Co. Ltd., Wuhan, China) in a CFX Connect Real-Time PCR System (Bio-Rad, Hercules, CA, USA). The 2^−ΔΔCt^algorithm was utilized to calculate the gene expression, which was normalized to GAPDH. All experiments were conducted in triplicate. The primer sequences were: PTEN, forward, 5’-CGTGCGGATAATGACAAGGA-3’ and reverse, 5’-GGATTTGATGGCTCCTCTACTG-3’; GAPDH, forward, 5’-CTGGAGAAACCTGCC AAGTATG-3’ and reverse, 5’-GGTGGAAGAATGGGAGTTGCT-3’.

### Immunohistochemical staining

Two groups of rat right femurs were fixed in 4% paraformaldehyde for 2 days, sliced into four mm-thick sections, and placed on sterile slides. These sections were permeabilized with 0.1% Triton X-100 for 15 min and deparaffinized for antigen retrieval. Nonspecific antigen binding was blocked by incubation with 5% normal goat serum for 30 min. Thereafter, the sections were incubated overnight at 4 °C with 1:1,000-diluted primary antibodies against phosphatase and tensin homolog deleted on chromosome 10 (PTEN) (Wuhan Servicebio Technology Co. Ltd., Wuhan, China). Thereafter, the sections were incubated with secondary HRP-labeled Goat Anti-Rabbit IgG (Wuhan Servicebio Technology Co. Ltd., Wuhan, China), and the nuclei were stained with hematoxylin. The sections were viewed using a white-light microscope, and then the Aipathwell software was applied to calculate the ratio of positive cells. The presence or absence of intracellular tan particles indicated positive and negative findings, respectively. Three fields of view per section were selected under ×20 magnification. Positive cells were counted, and average ratios were calculated. Ratios of positive cells were graded as 0 (0%–5%), 1 (6%–25%), 2 (26%–50%), 3 (51%–75%), or 4 >75% (Xie et al., 2014). The immunohistochemical staining results were analyzed using unpaired t-tests, and positive cell ratios (%) were calculated as:

\begin{eqnarray*}\text{Postive cell ratio}= \frac{\text{number of positive cells}}{\text{total number of cells}} . \end{eqnarray*}

### Validation of the core gene with ROC curves

The diagnostic accuracy of the hub gene was analyzed by ROC curves in the datasets GSE7429 and GSE56116 *via* GraphPad Prism9.5.1 software.

### GeneMANIA analysis and gene set enrichment analysis (GSEA)

GeneMANIA (http://www.genemania.org), a database for creating protein-protein interaction networks, was utilized to produce the network of core gene (Warde-Farley et al., 2010). In addition, single-gene GSEA was conducted to identify the vital roles and pathways of key gene involved in PMOP (Subramanian et al., 2005). *p*-Value <0.05 and —NES—>1 were selected as the criterion.

### TFs-mRNA-miRNAs network construction

The transcription factors (TFs)-mRNA interaction matches of the key gene were provided by TRRUST database (https://ngdc.cncb.ac.cn/databasecommons/database/id/5213) (Han et al., 2018), and the Sankey map was produced *via* the sangerbox website. The upstream miRNAs of the core gene were predicted by miRDB (https://mirdb.org/) (Chen & Wang, 2020), TargetScan (https://www.targetscan.org/) (Agarwal et al., 2015), miRWalk (http://mirwalk.umm.uni-heidelberg.de/) (Dweep, Gretz & Sticht, 2014), and miRTarBase (https://mirtarbase.cuhk.edu.cn/ miRTarBase/miRTarBase_2025/php/index.php) databases (Huang et al., 2020), respectively, and the intersection miRNAs were selected out by Venn plot. Finally, the TFs, miRNAs, and core gene were merged to form a regulatory network using Cytoscape software.

### Gene-disease correlations and gene-medication associations

DisGeNET (https://www.disgenet.org/) (Pinero et al., 2017), a comprehensive platform integrating information on human disease-associated genes, was used to identify novel perspectives on the relationships between the hub gene and disease, and the interactions were presented using the Cytoscape software. The drug-gene interaction database (DGidb) (https://www.dgidb.org) (Wagner et al., 2016) was utilized to predict therapeutic medications that interact with the hub gene. To analyze the probable therapeutic mechanisms of these medications, we conducted a molecular docking analysis on the key gene with the drugs. Firstly, the 3D structure of the drugs was downloaded from the PubChem database (https://pubchem.ncbi.nlm.nih.gov/) (Wang et al., 2012). The protein data bank (PDB) database (https://www.rcsb.org/) then provided us with the protein crystal structure of the hub gene (Burley et al., 2017). Finally, we conducted a gene-drug docking analysis based on the AutoDock software (version, 4.2.6), and the PyMOL software (version, 3.1) was used for visualization.

### Immune infiltration analysis

To assess the infiltration of 22 kinds of immune cells and estimate the roles of the immune microenvironment in PMOP progression, the immune infiltration differences between PMOP and control groups were evaluated by CIBERSORTx (https://cibersortx.stanford.edu/). Furthermore, associations between the hub gene and 22 immunocytes were identified by utilizing Pearson correlation analysis. All visualizations were created through the sangerbox platform.

### Statistical analysis

All data were statistically analyzed using GraphPad Prism 9.5.1 (GraphPad Software Inc., San Diego, CA, USA) and SPSS 25.0 (IBM Corp., Armonk, NY, USA). All experiments were repeated at least three times to validate the results, and the data are presented as means ± standard deviation. Between-group differences were compared using Student unpaired t-tests. Values with a *P* < 0.05 were deemed statistically significant. *P < 0.05, **P < 0.01, ***P < 0.001, ****P < 0.0001.

## Results

### Identification of DEGs and FRGs

**Figure 2:**
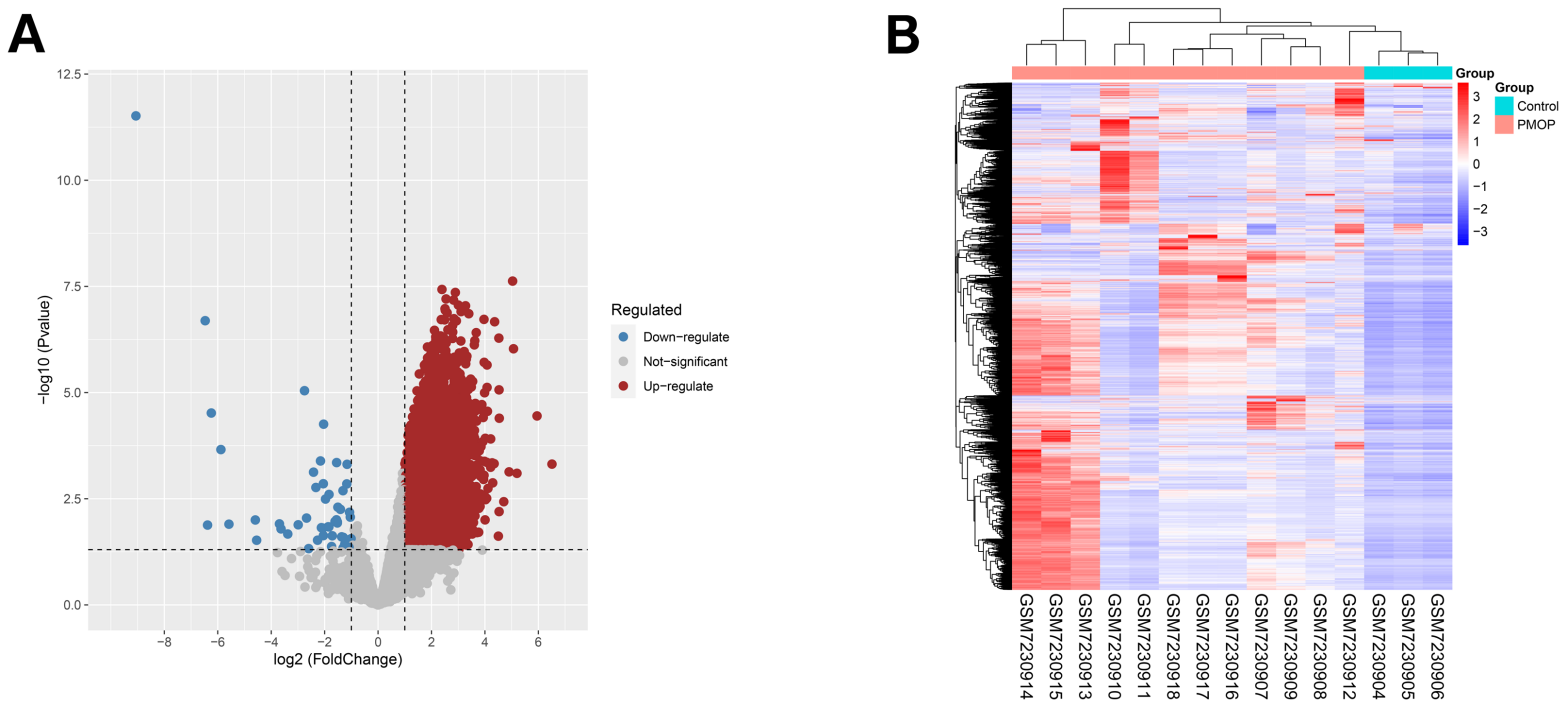
Identification of differentially expressed genes (DEGs) in the GSE230665 dataset. (A) A volcano plot showing DEGs in the GSE230665 dataset. (B) Heatmap of DEGs.

GSE230665 is a novel PMOP dataset and we acquired 12,422 DEGs by the limma method, comprising 12,372 upregulated and 50 downregulated DEGs, which was shown by a volcano and a heatmap (Figs. 2A, 2B). Then, we acquired 484 FRGs (Table S2) from the FerrDb database after removing the duplicate ones.

### Weighted gene co-expression network analysis and significant module identification

**Figure 3:**
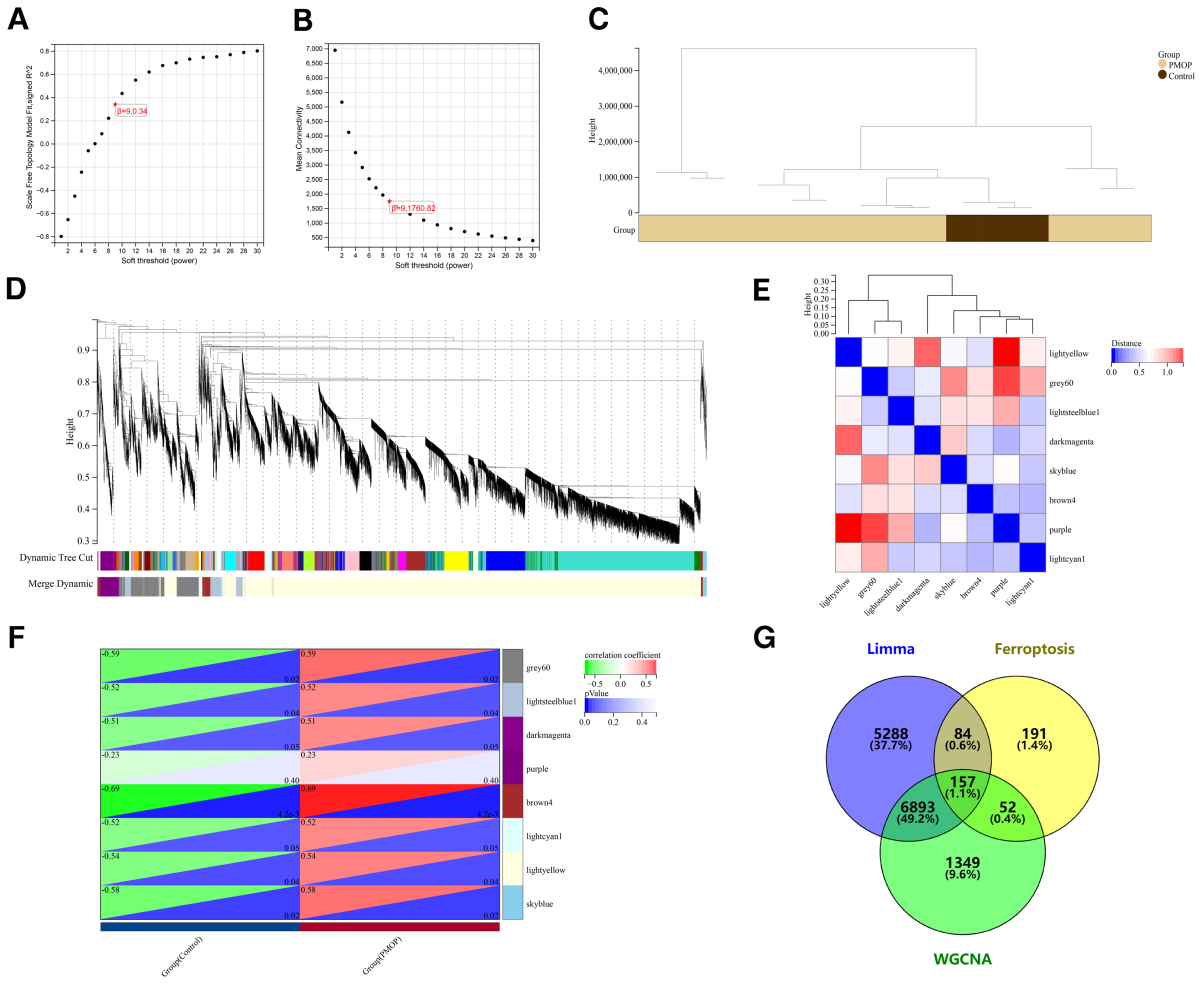
Identification of FDEGs and module genes in PMOP. (A, B) The soft threshold *β* = 9 is set based on scale independence and average connectivity. (C) Clustering dendrogram of the PMOP and control groups. (D) The gene tree depicts gene modules, which are visually distinguished by various colors. (E) Heatmap of eigengene adjacency. (F) The heatmap reveals that the brown4 module exhibits the strongest relevance with PMOP. The bottom right triangle in each pair is the *p*-value, while the top left triangle represents the correlation coefficient. (G) A total of 157 FDEGs were screened out based on the Venn diagram.

We established a scale-free co-expression network *via* the WGCNA to determine the most relevant module in PMOP. A “soft” threshold *β* = 9 was selected based on scale independence and average connectivity (Figs. 3A, 3B). The clustering dendrogram of the PMOP and control groups is depicted in Fig. 3C. By setting the module merge cutoff of 0.25, sensitivity of 3, and a minimum size of 30, we finally acquired 8 modules with various colors (Figs. 3D, 3E), and the brown4 module exhibited the strongest association with PMOP (correlation coefficient =0.69, *p*-value <0.01) (Fig. 3F). Then, the genes in the top 4 modules with the highest correlation coefficient (brown4, grey60, skyblue, and lightyellow) were considered significant and extracted for further analysis, and a total of 8,451 module genes were obtained. Next, we intersected the DEGs, FRGs, and module genes, and finally acquired 157 FDEGs (Fig. 3G).

### Functional enrichment analysis of FDEGs

**Figure 4:**
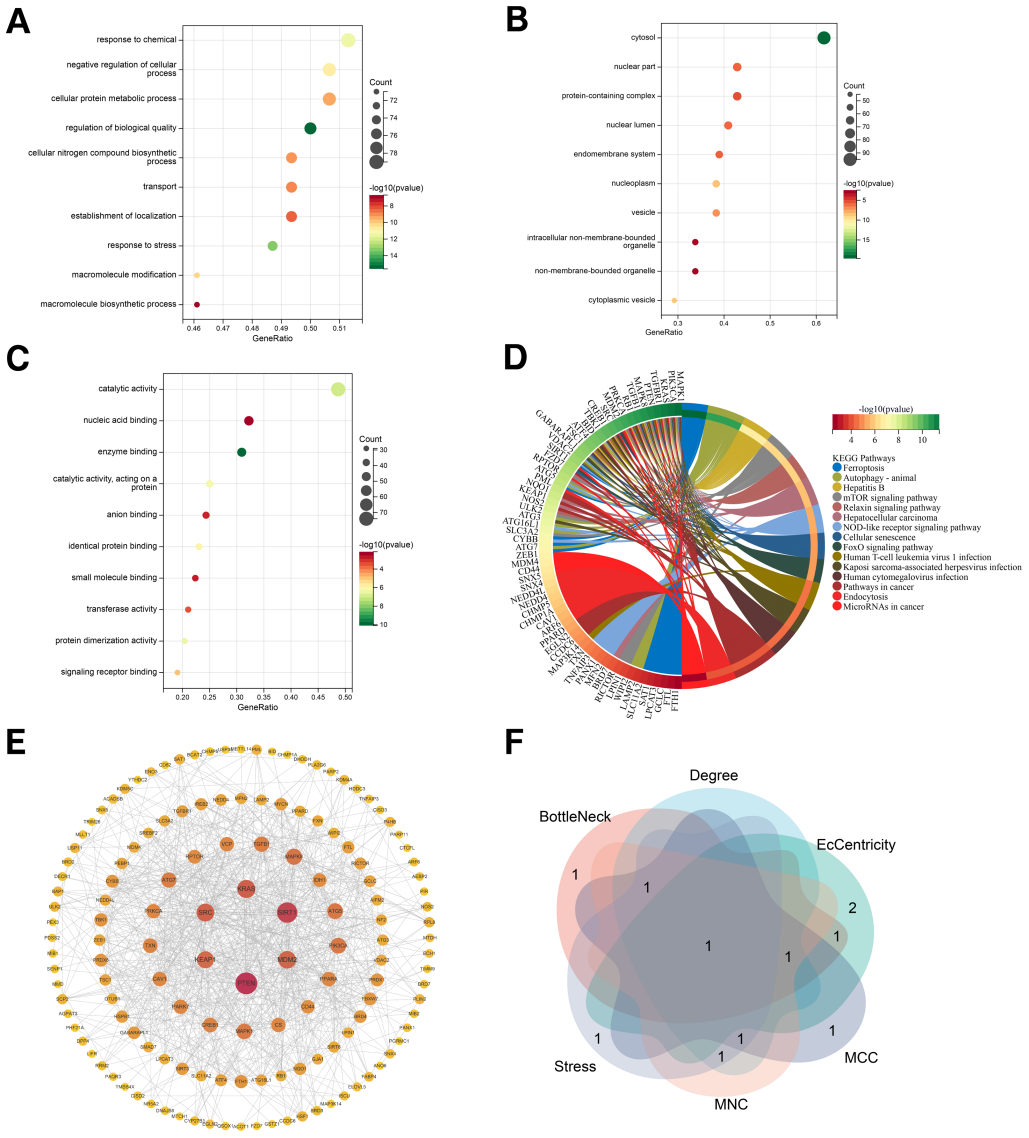
Functional enrichment analyses of FDEGs. The bubble graph shows BP (A), CC (B), and MF (C). (D) Circle plot of KEGG pathway enrichment analysis. (E) A PPI network containing 143 nodes and 767 edges. (F) The core gene PTEN was identified through six algorithms.

To further explore the functions and pathways involved in FDEGs, GO, and KEGG enrichment analysis were executed by the“clusterProfiler” package. The results suggested that the genes were enriched in BP associated with response to chemical, negative regulation of the cellular process, cellular protein metabolic process, and transport (Fig. 4A). Genes enriched in cellular components were predominantly localized in the cytosol, nuclear part, protein-containing complex, and nuclear lumen (Fig. 4B). Genes enriched in molecular functions were mostly clustered on catalytic activity, nucleic acid binding, enzyme binding, and anion binding (Fig. 4C). The KEGG results revealed that the signaling pathways of cancer, autophagy-animal, hepatitis B, mTOR, endocytosis, and ferroptosis were significant (Fig. 4D).

### Construction of the PPI network and identification of the core gene

We analyzed the PPI network of FDEGs using the STRING database and then hid unconnected nodes in the network, as 14 of the 157 genes were unrelated to the others. The final PPI network contained 143 nodes and 767 edges (Fig. 4E). The results of the MCODE analysis revealed components with extremely densely packed clusters, resulting in 5 groups (Table 1). The six algorithms in cytoHubba, namely Degree, Stress, BottleNeck, MCC, MNC, and EcCentricity, were utilized to compute the top five genes respectively from the PPI network (Fig. S1), and then they were intersected to generate the core gene PTEN based on a Venn diagram (Fig. 4F).

### Verification of the top genes *in vivo*

**Figure 5:**
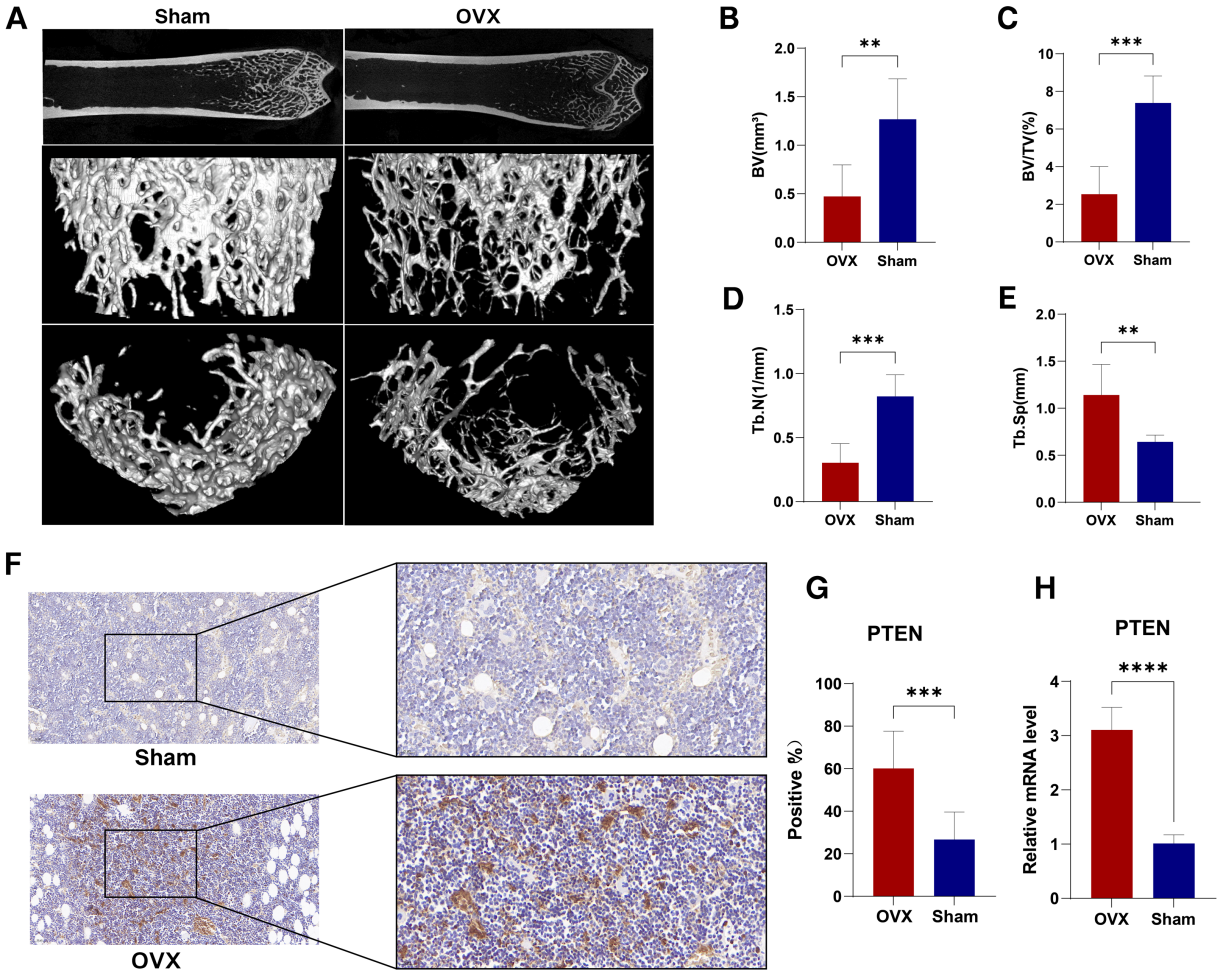
Micro-computed tomography (micro-CT) analysis of tibia and experimental verification. (A) Representative micro-CT images of tibias and three-dimensional reconstruction images from experimental group (OVX, *n* = 6) or control group (Sham, *n* = 6) rats. (B–E) Quantitation of trabecular BV, BV/TV, Tb. N, and Tb. Sp. (F–G) Representative images of immunohistochemically stained femur tissues and quantitation of positive expression of PTEN. (H) qRT-PCR results for PTEN expression. The immunohistochemical staining and qRT-PCR of PTEN were repeated three times respectively and the data are presented as means ± standard deviation. Left and right scale bars: 50 µm. *p < 0.05, **p < 0.01, ***p < 0.001, ****p < 0.0001.

We removed bilateral ovaries from three SD rats to develop a model of PMOP *in vivo*, and the rats were euthanized 12 weeks after surgery with a pentobarbital overdose, and the bilateral tibias were removed for micro-computed tomography (micro-CT) analysis (Fig. 5A). The BV, BV/TV, Tb. N were considerably lower, whereas Tb. Sp was higher in the OVX group compared to the sham-operated group, indicating that the rats had developed PMOP (Figs. 5B, 5E). We then analyzed the mRNA and protein levels for PTEN expression in the OVX and sham-operated rats. IImmunohistochemical staining unveiled that compared with the sham-operated group, the expression level of PTEN in the OVX group was significantly higher (Figs. 5F, 5G), and the same result was also obtained by qRT-PCR analysis (*P* < 0.05) (Fig. 5H). This finding may suggest a potential correlation between PMOP and PTEN.

**Table 1:** Details of five clusters.

Cluster	Score	Nodes	Edges	Genes
1	8.235	18	70	PTEN, CREB1, MDM4, PIK3CA, MDM2, NF2, SRC, CD44, ZEB1, RB1, SIRT1, BRD4, HSPB1, NEDD4, TGFB1, TGFBR1, PEBP1, LPCAT3
2	6.737	20	64	ATG16L1, ATG3, WIPI2, TBK1, GABARAPL1, ULK2, RPTOR, MAPK8, KEAP1, PPARA, PRKCA, TSC1, CYBB, KRAS, MAPK1, IDH1, RICTOR, CAV1, FBXW7, MYCN
3	3.273	12	18	FABP4, PPARD, PLIN2, SREBF2, SIRT6, NQO1, GCLC, FTL, FXN, PLA2G6, PRDX6, ISCU
4	3	11	15	AIFM2, SLC11A2, IREB2, SLC3A2, SAT1, PARK7, P4HB, PRDX1, MFN2, ATG7, ATG5
5	3	3	3	RRM2, HDDC3, CS

### Validation of hub gene from the GSE7429 and GSE56116 datasets

**Figure 6:**
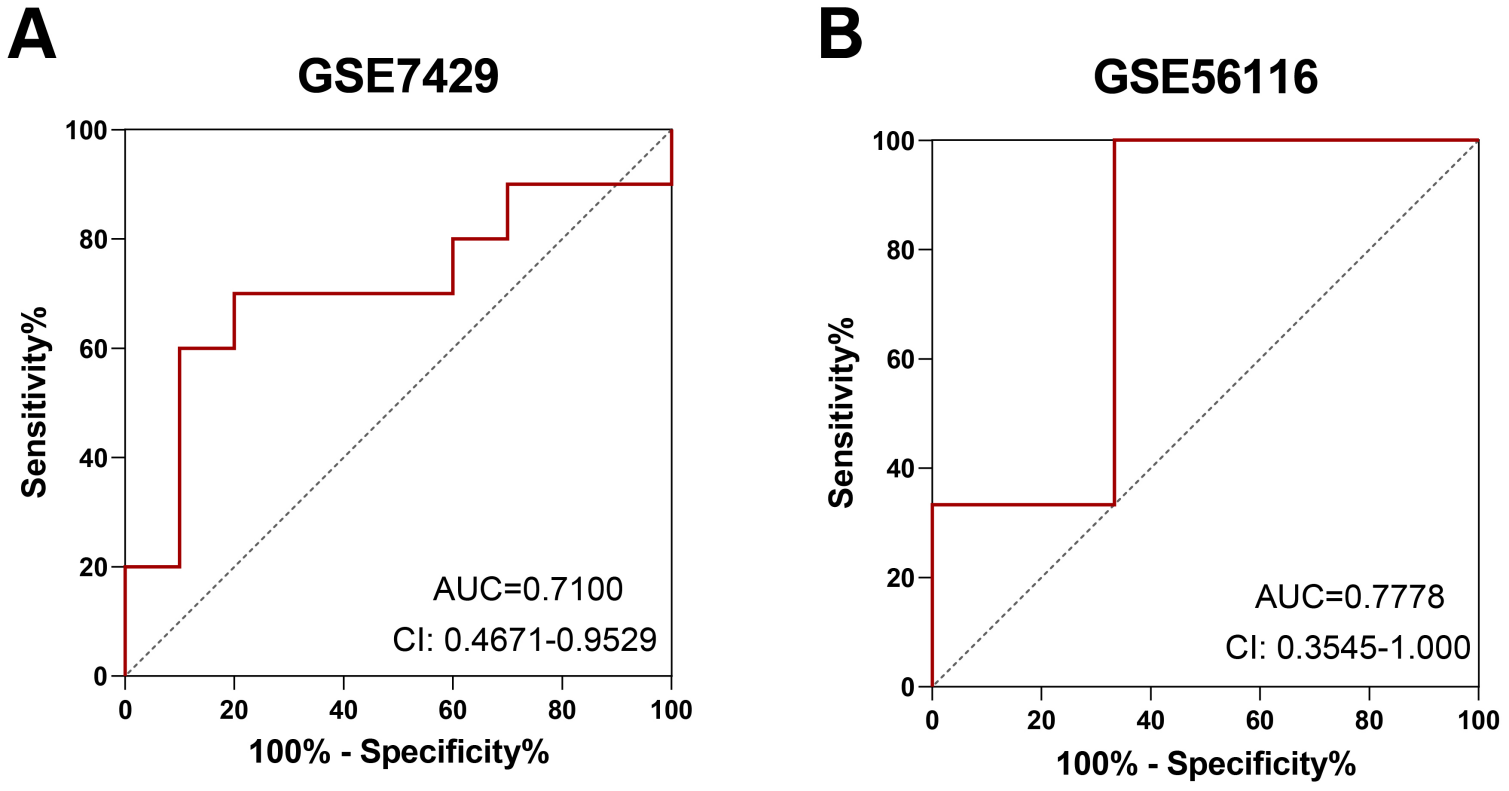
ROC analysis of PTEN: (A) GSE7429 dataset. (B) GSE56116 dataset.

We downloaded another two datasets to verify the differential expression of PTEN, thus the ROC examination of PTEN was carried out on the GSE7429 and GSE56116 datasets (Table S3). It showed that the area under curves (AUCs) of PTEN was beyond 0.7, indicating its potential diagnostic power for PMOP (Figs. 6A, 6B).

### GeneMANIA analysis and single-gene GSEA of PTEN

**Figure 7:**
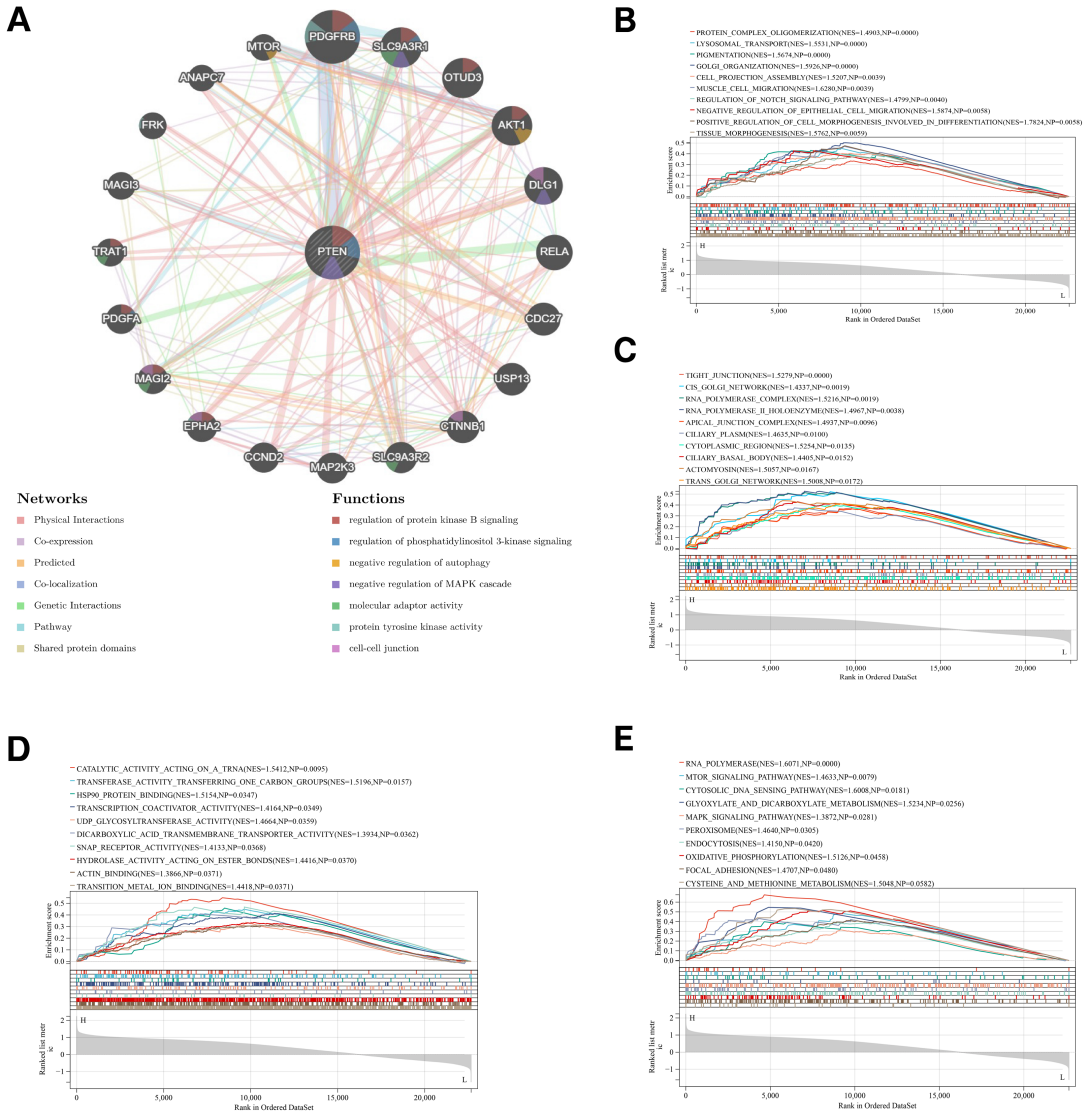
GeneMANIA analysis and single-gene GSEA of PTEN. (A) The PPI network of PTEN. The GSEA analysis includes biological process (B), cellular component (C), molecular function (D), and KEGG pathway (E) for PTEN.

Figure 7A illustrates a PPI network and associated functions of PTEN. GeneMANIA study proved that these mechanisms were primarily related to the protein tyrosine kinase activity, molecular adaptor activity, and negative regulation of MAPK cascade, *etc*. Single-gene GSEA was further performed for better comprehending the roles and signaling pathways engaged in PTEN. Totally, 1,076 GO items including BP, CC, and MF (Figs. 7B–7D), and 22 KEGG pathways (Fig. 7E) of PTEN were evaluated, and we presented 10 important GO terms and KEGG pathways, namely protein complex oligomerization, RNA polymerase complex, transcription coactivator activity, MTOR signaling pathway, *etc*.

### TFs-mRNA-miRNAs network construction

**Figure 8:**
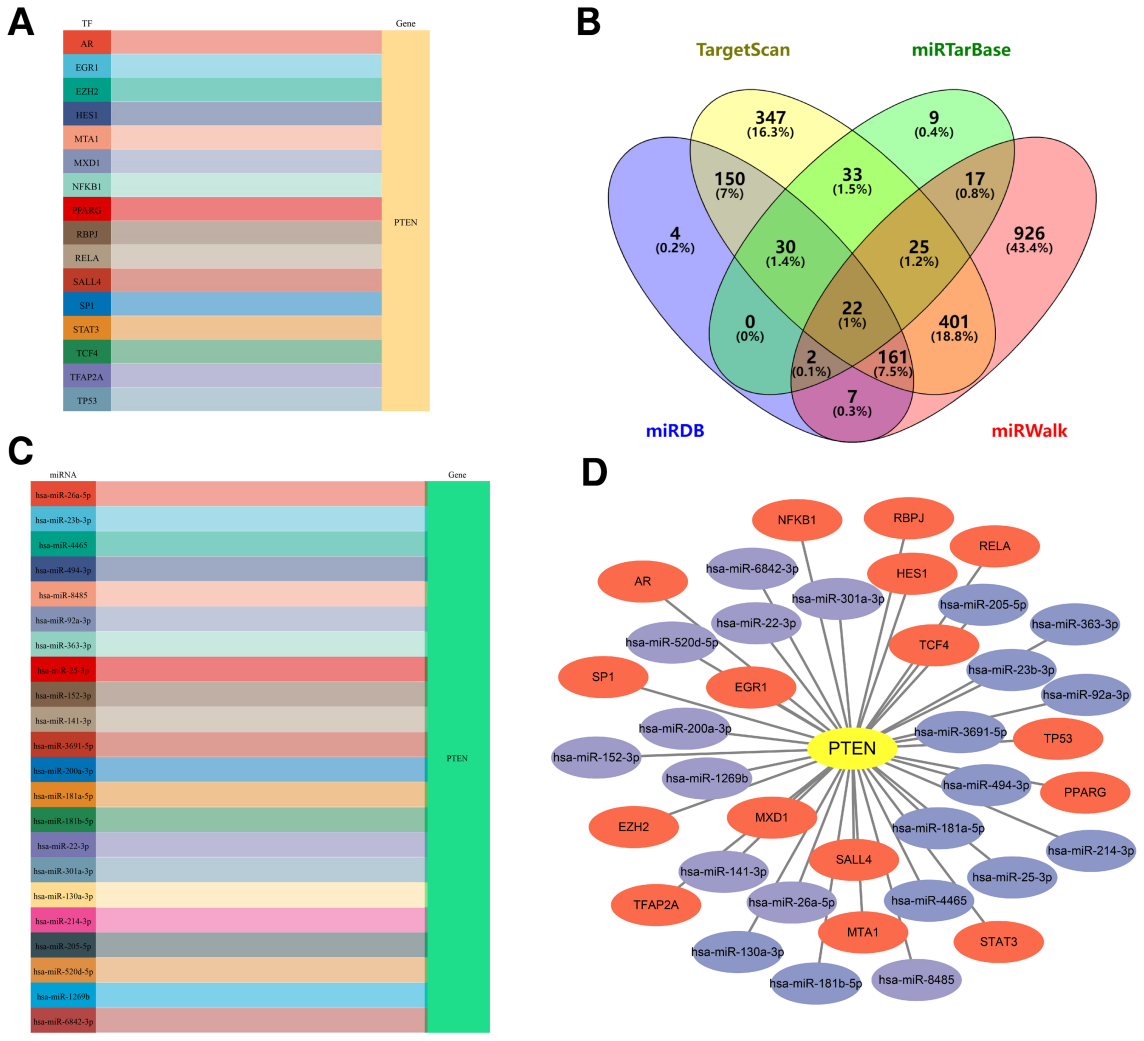
TFs-mRNA-miRNAs network of PTEN. (A) Sankey diagram illustrates the 16 interaction pairs of TF-mRNA from PTEN. (B) Twenty-two shared miRNAs were screened out by the Venn diagram. (C) miRNAs-mRNA regulatory graph. (D) TFs-mRNA-miRNAs connection network.

At first, 16 interaction pairs of TF-mRNA were detected from the TRRUST database based on PTEN (Fig. 8A), and the 22 mutual miRNAs were extracted from 4 different databases mentioned above (Fig. 8B). Next, the miRNAs-mRNA connection diagram was constructed according to the 22 miRNAs and 1 core gene (Fig. 8C). Finally, all TFs, miRNAs, and mRNA were combined to establish the TFs-mRNA-miRNAs regulatory network, including 39 nodes (16 TFs, 22 miRNAs, and 1 mRNA) and 38 edges (Fig. 8D), in which AR, EGR1, and EZH2, hsa-miR-152-3p, hsa-miR-8485, hsa-miR-363-3p, *etc*. regulated PTEN. However, these results require further confirmation.

### Gene-disease correlations and gene-medication docking analysis

**Figure 9:**
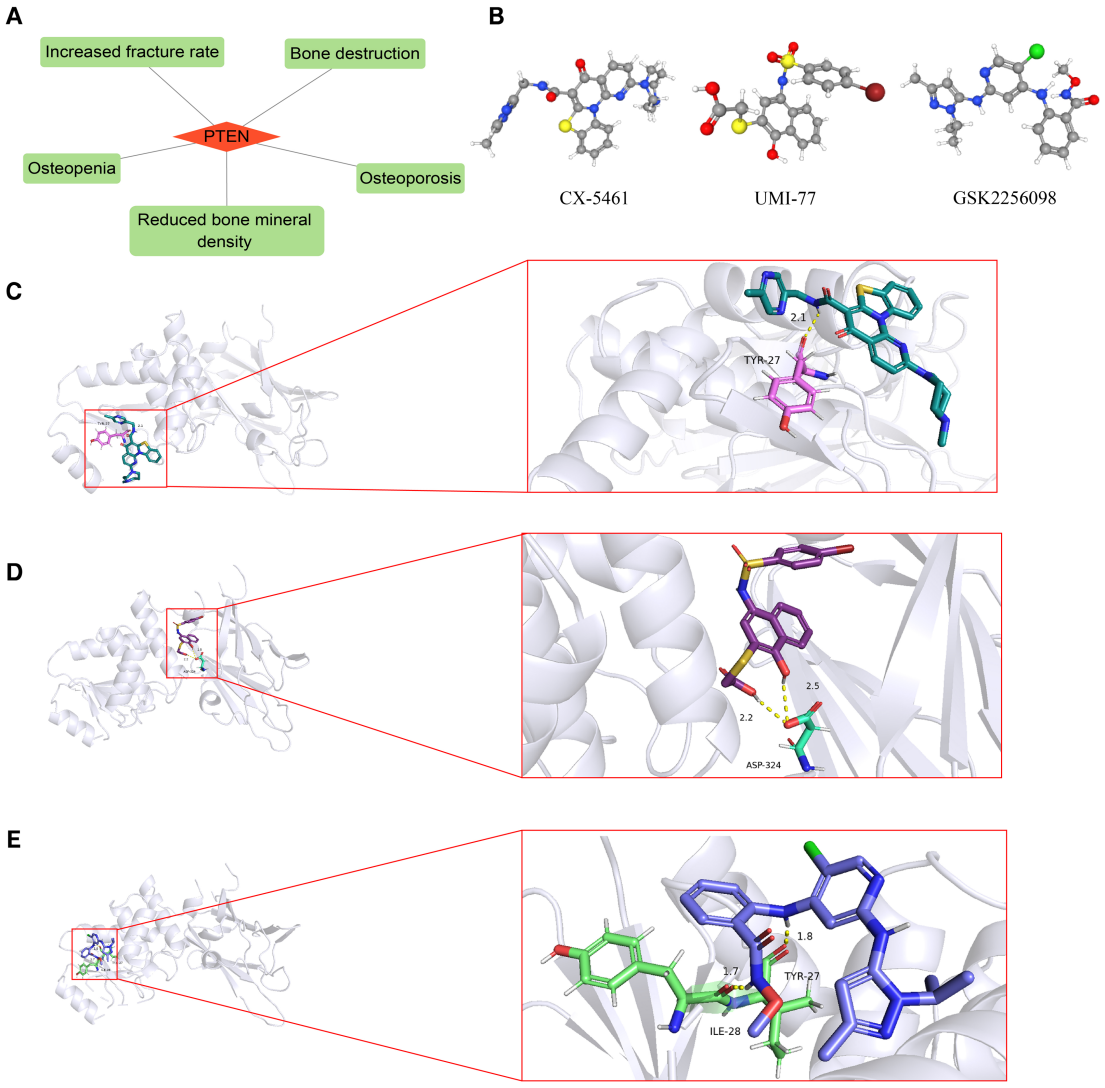
Gene-disease correlations and gene-medication associations. (A) Gene-disease interactions. (B) The 3D structures of CX-5461, UMI-77, and GSK2256098. The docking relationships among PTEN and the CX-5461(C), UMI-77 (D), and GSK2256098 (E).

To investigate the disorders that PTEN is involved in, we adopted the DisGeNET database for gene-disease connections and discovered that the pertinent diseases contained reduced bone mineral density, osteoporosis, osteopenia, increased fracture rate, and bone destruction (Fig. 9A). Potential therapeutic drugs interacting with PTEN were found by applying the DGIdb database and the top 10 drugs were extracted based on the interaction score (Table 2). Subsequently, we chose the top three medicines (CX-5461, UMI-77, and GSK2256098) for gene-drug docking analysis to explore the potential therapeutic mechanisms of the drugs, and the 3D structures of these three medications were retrieved *via* the PubChem database (Fig. 9B). Additionally, the protein crystal structure of PTEN (PDBID: 7JTX) was obtained from the PDB database. The docking analysis discovered that the binding energies of PTEN protein to CX-5461, UMI-77, and GSK2256098 were −6.0 kcal-mol-1, −3.51 kcal-mol-1, and −4.26 kcal-mol-1, respectively, indicating a good binding affinity among the three drugs and PTEN (Figs. 9C–9E).

### Immune cell infiltration analysis in PMOP

**Figure 10:**
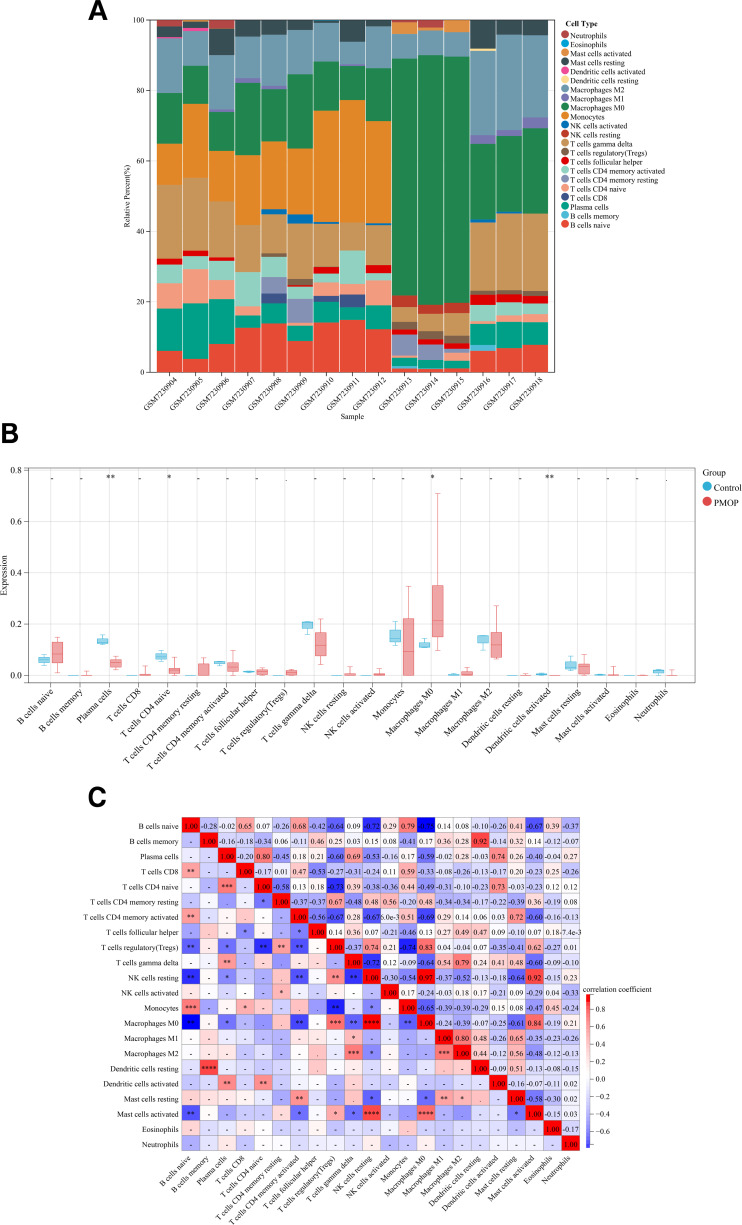
Immune cell infiltration analysis between PMOP and control groups. (A) The bar-plot showed the composition of 22 kinds of immune cells in GSE230665. (B) The difference in immune infiltration of 22 types of immune cells was shown by a box-plot. (C) Correlation of 22 kinds of immune cells. *, *p* < 0.05, **, *p* < 0.01, ***, *p* < 0.001.

**Figure 11:**
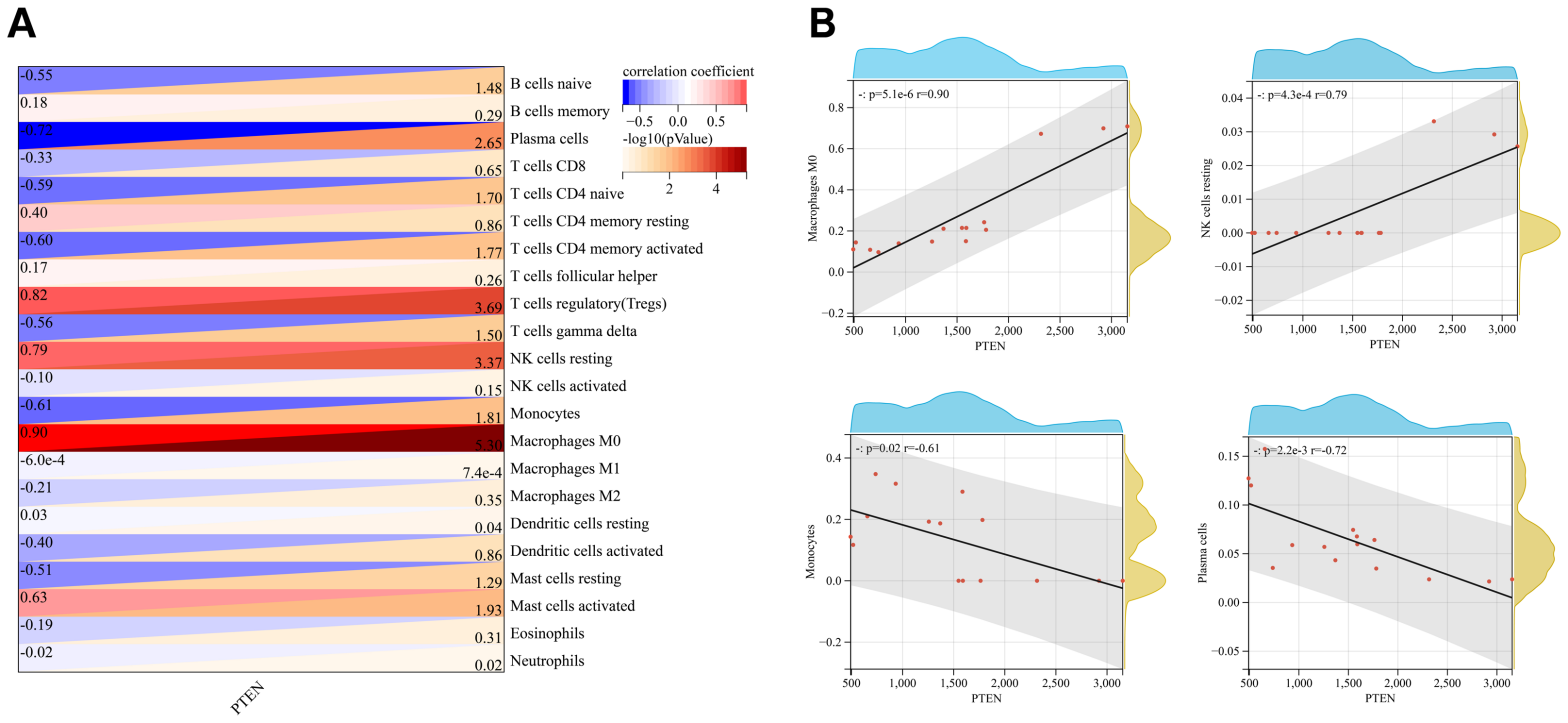
Associations between PTEN and immune cells. (A) Pearson correlation analysis between PTEN and 22 types of immunocytes. Red: positive correlation; blue: negative correlation. (B) Four kinds of immune cells with positive or negative correlation with PTEN were listed.

Considering the relationship between PMOP and chronic inflammatory microenvironment *in vivo*, the 22 kinds of immune cells that participated in the development of PMOP were analyzed by CIBERSORTx. We eventually acquired the relative percent of immune cells between the PMOP and control groups (Fig. 10A). In comparison with the control group, the proportions of macrophages M0 were higher, whereas plasma cells, T cells CD4 naive, and dendritic cells activated were lower in the PMOP group, and the differences were statistically significant (Fig. 10B; Table S4). The correlation of 22 species of immune cells suggested that resting NK cells were positively related to M0 macrophages (*r* = 0.97) and activated mast cells (*r* = 0.92), while naive B cells were negatively associated with M0 macrophages (*r* =  − 0.75) (Fig. 10C). Additionally, the correlations between PTEN and 22 immunocytes were evaluated by applying Pearson correlation analysis (Fig. 11A). PTEN exhibited a positive correlation with M0 Macrophages (*r* = 0.90 and *p* < 0.05) and resting NK cells (*r* = 0.79 and *p* < 0.05), while negatively correlated with Monocytes (*r* =  − 0.61 and *p* < 0.05) and plasma cells (*r* =  − 0.72 and *p* < 0.05) (Fig. 11B). In summary, multiple immune cells were differently infiltrated in PMOP patients, which might act as a regulatory target for PMOP intervention.

## Discussion

OP is a frequent chronic metabolic bone disease among elderly persons, and it is categorized as primary (type I, type II, and idiopathic) and secondary etiological types. Type I generally develops in postmenopausal women, and type II usually appears after the age of 70 years. However, the cause of idiopathic OP is unclear, and it often affects younger people. Secondary OP is typically triggered by some diseases, medications, or other factors, and it is prevalent in men and premenopausal women (Jin et al., 2020; Li et al., 2021). Ferroptosis is intricately linked to glucocorticoid-induced and diabetic OP, as well as PMOP (D’Amelio et al., 2008; Yang et al., 2021). Inhibiting the ferroptosis pathway confers considerable therapeutic effects on the deteriorative bone microstructure of OVX mice (Liu et al., 2022a). Improved early screening is urgently needed to recognize risk factors for OP and actively implement preventive measures because postmenopausal women are particularly susceptible to OP, which is frequently missed due to its subtle onset (Liu et al., 2022b). Therefore, we applied a bioinformatic approach to determine which ferroptosis genes correlate most closely with PMOP and verified their expressions in the PMOP rat model.

**Table 2:** The potential top 10 drugs associated with PTEN.

Gene	Drug	Interaction score
PTEN	CX-5461	0.6914410405150402
PTEN	UMI-77	0.6914410405150402
PTEN	GSK2256098	0.6914410405150402
PTEN	IPATASERTIB	0.4085787966679783
PTEN	MULTI-AGC KINASE INHIBITOR AT13148	0.3457205202575201
PTEN	RISOVALISIB	0.3457205202575201
PTEN	BORUSSERTIB	0.3457205202575201
PTEN	CHEMBL:CHEMBL589258	0.3457205202575201
PTEN	GDC-0152	0.3457205202575201
PTEN	MEK INHIBITOR CI-1040	0.3457205202575201

We performed PPI analysis and the hub gene, namely PTEN, was screened out from the PPI network based on six algorithms. PTEN, the first tumor suppressor gene to be recognized as having double specificity, expresses a lipid and protein phosphatase (Chen et al., 2018). Bone morphogenetic protein 9 (BMP9) is a powerful, effective inducer of osteogenic stem cell differentiation (Liu et al., 2020). Bone marrow mesenchymal stem cells (BMMSCs) differentiation into osteoblasts induced by BMP9 is inhibited by PTEN. Silencing PTEN activates the Wnt/*β*-catenin pathway in BMMSCs, which boosts their osteogenic differentiation, thus considerably attenuating OP in OVX mice (Li et al., 2020; Shen et al., 2020). Here, GSEA enrichment analysis of PTEN showed 1076 GO terms (transcription coactivator activity, hsp90 protein binding and lysosomal transport, *etc.*) and 22 KEGG pathways (MAPK signaling pathway, MTOR signaling pathway, oxidative phosphorylation, *etc*.). Li et al. (2022) confirmed that melatonin (MT) can suppress ferroptosis by activating the PI3K/AKT/mTOR signaling pathway, thus avoiding the emergence of steroid-induced osteoporosis. Another major feature of ferroptosis is the abundant accumulation of intracellular ROS, which ultimately induces cell damage and death (Park & Chung, 2019). Isosinensetin could decrease osteoclastogenesis and bone absorption in OVX mice by inhibiting the ROS-mediated MAPK signaling pathway, implying that the hub gene PTEN may participate in PMOP progression and contribute to bone remodeling imbalances by regulating ferroptosis (Qin et al., 2023).

MiRNA can carry out post-transcriptional regulation by partly combining with the target mRNA. Meanwhile, TF, as the upstream regulator, can mediate the expression of mRNA, thus contributing to a complicated TFs-mRNA-miRNAs co-regulatory system (Ye et al., 2020; Fu et al., 2021). TFs-mRNA-miRNAs network could assist in connecting the relationships of TF, miRNA, and target gene, thereby researching potential significant components more efficiently and intuitively (Luo et al., 2022; Ke et al., 2021). In our research, the TFs-mRNA-miRNAs network revealed that PPAGR, EGR1 and RELA, hsa-miR-363-3p, hsa-miR-92a-3p, hsa-miR-214-3p, *etc*. regulated PTEN. Li et al. (2019) reported that miR-363-3p suppresses osteogenic differentiation and promotes osteoclastogenesis by targeting PTEN to activate the PI3K/AKT signaling pathway, thereby aggravating OP. MiR-92a-3p could enhance osteogenesis and reverse OP in mice *via* the PTEN/AKT signaling pathway (Xu et al., 2023). Additionally, miR-214 suppresses osteoclast differentiation and mitigates OP in OVX mice by inhibiting the PI3K/AKT/NF-*κ*B signaling pathway (Wang et al., 2020). So far, the precise mechanism of numerous miRNAs in PMOP remains unclear. We have discovered the key gene, PTEN, as well as creating a core gene-related TFs-mRNA-miRNAs network. This may greatly promote further research on the pathogenesis and act as a possible biomarker for PMOP diagnosis and treatment.

Furthermore, we established OVX rat models of PMOP to validate our results. QRT-PCR analysis and immunohistochemical staining revealed increased PTEN expression in femoral tissues from the OVX compared with those from the sham-operated rats. Meanwhile, the ROC curves of the GSE7429 and GSE56116 datasets revealed that the AUCs of PTEN have relatively high efficiency in the diagnosis of PMOP, suggesting that PTEN may play crucial roles in PMOP pathogenesis. Consequently, our findings may provide another worthwhile approach to elucidating PMOP pathogenesis.

So far, the definite relationship between ferroptosis and immunity is elusive and needs to be further investigated. Thereby, we employed CIBERSORTx to research the infiltration of immune cells in the femoral tissues of PMOP. The results demonstrated that compared with the control group, the proportions of macrophages M0 were relatively high, while plasma cells, T cells CD4 naive, and dendritic cells activated were relatively low in PMOP. Nonetheless, our study had some limitations. We did not intervene with ferroptosis inhibitors in OVX rats and ignored important procedures such as measuring ferroptosis indicators in rat bone tissues.

## Conclusion

Altogether, we identified and validated ferroptosis-associated key gene in PMOP using bioinformatic methods, which provided a theoretical foundation for further deeper explorations. We are preparing to gather sufficient bone tissue from clinical patients with PMOP to further validate the present findings and render our conclusions more credible.

##  Supplemental Information

10.7717/peerj.20666/supp-1 Supplemental Information 1 Summary of patients’ parameters

10.7717/peerj.20666/supp-2 Supplemental Information 2 Ferroptosis-related genes (FRGs)

10.7717/peerj.20666/supp-3 Supplemental Information 3 The ROC curves of PTEN was plotted using the GSE56116 and GSE7429 datasets

10.7717/peerj.20666/supp-4 Supplemental Information 4 The proportion of 22 kinds of immune cell types in GSE230665 dataset

10.7717/peerj.20666/supp-5 Supplemental Information 5 Bioinformatics Code

10.7717/peerj.20666/supp-6 Supplemental Information 6 Aipathwell immunohistochemical analysis report of PTEN

10.7717/peerj.20666/supp-7 Supplemental Information 7 Supplementary Figure s1

10.7717/peerj.20666/supp-8 Supplemental Information 8 qRT-PCR steps

10.7717/peerj.20666/supp-9 Supplemental Information 9 All figures raw data

10.7717/peerj.20666/supp-10 Supplemental Information 10 MIQE checklist

10.7717/peerj.20666/supp-11 Supplemental Information 11 ARRIVE guidelines

## Additional Information and Declarations

### Data Availability

The following information was supplied regarding data availability: The raw data and code are available in the Supplemental Files.
