# Supplementary material for: Identification and verification of ferroptosis-related core gene in postmenopausal osteoporosis based on bioinformatics analysis
Source: PeerJ. 2026 Mar 31;14:e20666. doi: 10.7717/peerj.20666 (PMC13048226; doi:10.7717/peerj.20666)
Supplement: Supplemental Information 1 [file peerj-14-20666-s001.docx]

Supplementary Table S1. Summary of patients’ parameters

| Group | Postmenopausal osteoporosis group | control group |
| --- | --- | --- |
| Donors | 12 | 3 |
| Average age (years) | 61.92±4.94 | 64.33±3.06 |
| Gender | 12*female | 3*female |
| Tissue | Femur(1g) | Femur(1g) |
