# Supplementary material for: Identification and verification of ferroptosis-related core gene in postmenopausal osteoporosis based on bioinformatics analysis
Source: PeerJ. 2026 Mar 31;14:e20666. doi: 10.7717/peerj.20666 (PMC13048226; doi:10.7717/peerj.20666)
Supplement: Supplemental Information 2 [file peerj-14-20666-s002.docx]

Supplementary Table S2

Ferroptosis-related genes (FRGs)

| Ferroptosis driver | Ferroptosis suppressor | Ferroptosis marker |
| --- | --- | --- |
| RPL8, IREB2, ATP5MC3, CS, EMC2, ACSF2, NOX1, CYBB, NOX3, NOX4, NOX5, DUOX1, DUOX2, G6PD, PGD, VDAC2, PIK3CA, FLT3, SCP2, TP53, ACSL4, LPCAT3, NRAS, KRAS, HRAS, TF, TFRC, TFR2, SLC38A1, SLC1A5, GLS2, GOT1, CARS1, ALOX5, KEAP1, HMOX1, ATG5, ATG7, NCOA4, ALOX12, ALOX12B, ALOX15, ALOX15B, ALOXE3, PHKG2, ACO1, ATG4D, BECN1,GABARAPL2,ATG16L1, WIPI1, WIPI2, SNX4, ATG13, ULK2, SAT1, EGFR, MAPK3, MAPK1, BID, GABARAPL1, ZEB1, DPP4, CDKN2A, PEBP1, SOCS1, CDO1, MYB, MAPK8, MAPK9, CHAC1, MAPK14, LINC00472, PRKAA2, PRKAA1, MAP1LC3A, ELAVL1, BAP1, ABCC1, MIR6852, ACVR1B, TGFBR1, EPAS1, HILPDA, HIF1A, IFNG, ANO6, LPIN1, HMGB1, TNFAIP3, TLR4, ATF3, ATM, YY1AP1, EGLN2, MIOX, TAFAZZIN, MTDH, IDH1, SIRT1, FBXW7, PANX1, DNAJB6, BACH1, LONP1, CD82, IL1B, CTSB, POR, CYB5R1, ELOVL5, FADS1, PTEN, NR1D1, NR1D2, TBK1, IL6, USP7, miR-182-5p, miR-378a-3p, ATF4, AQP3, AQP5, AQP8, LINC00618, MT1DP, PEX10, AGPAT3, PEX12, CHP1, GPAT4, BRPF1, OSBPL9, INTS2, MMD, CYP4F8, MLLT1, TTPA, GRIA3, EPT1, POM121L12, LIG3, AEBP2, AGPS, CDCA3, PEX2, PEX6, TIMM9, DCAF7, LCE2C, FAR1, PHF21A, SMAD7, LYRM1, AMN, PEX3, MTCH1, ACADSB, PVT1, hsa_circ_0008367, SLC39A14, MAP3K11, GSK3B, BRD7, SLC25A28, MFN2, SLC11A2, ZFAS1, TSC1, TGFB1, SNCA, SIRT3, CGAS, STING1, HDDC3, MIR761, MDM2, MDM4, MIR214, DLD, WWTR1, PRKCA, LGMN, SMPD1, MYCN, IFNA1, IFNA2, IFNA4, IFNA5, IFNA6, IFNA7, IFNA8, IFNA10, IFNA13, IFNA14, IFNA16, IFNA17, IFNA21, SMG9, PPARG, MIR335, SNX5, PAQR3, MICU1, TOR2A, MIR375, MAP3K14, CircKDM4C, MIR324, QSOX1, MIB2, CLTRN, KLF2, MIR5096, HOTAIR, H19, FOXO4, YTHDC2, DDR2, SLC39A7, TRIM46, ACSL1, KDM5A, TRIM21, DPEP1, CYGB, IDO1, GSTZ1, GJA1, SLC7A11, PGRMC1, CIRBP, circPSEN1, USP11, YAP1, MIR135B, TRIM26, NDRG1, MIR302A, ASMTL-AS1, FADS2, PIEZO1, LIFR, PTPN6, MIR15A, ADAM23, ARHGEF26-AS1,CPEB1, COX4I2, TIMP1, KDM6B, METTL14, CFL1, lncRNA AABR07017145.1, MIB1, KDM5C, MEG3, CCDC6, MIR539, KMT2D, EGR1, G6PDX, ULK1, ATG3 | SLC7A11, GPX4, AKR1C1, AKR1C2, AKR1C3, RB1, HSPB1, HSF1, GCLC, NFE2L2, SQSTM1, NQO1, HMOX1, FTH1, MUC1, SLC3A2, MT1G, SLC40A1, CISD1, FANCD2, FTMT, HSPA5, ATF4, TP53, HELLS, SCD, FADS2, SRC, STAT3, PML, MTOR, NFS1, TP63, CDKN1A, MIR137, ENPP2, VDAC2, FH, CISD2, MIR9-1, MIR9-2, MIR9-3, CBS, ISCU, ACSL3, OTUB1,CD44, LINC00336, BRD4, PRDX6, MIR17, SESN2, NF2, ARNTL, HIF1A, JUN, CA9, TMBIM4, PLIN2, MIR212, Fer1HCH, AIFM2, LAMP2, ZFP36, PROM2, CHMP5, CHMP6, CAV1, GCH1, SIRT3, DAZAP1 PIR, FTL, HCAR1, SLC16A1, RRM2, NR4A1, PIK3CA, RPTOR, SREBF1, SREBF2, FZD7, P4HB, NT5DC2, BCAT2, PLA2G6, MIR424, PARK7, FXN, SUV39H1, ATF2, ACOT1, ALDH3A2, STK11, FNDC5, CircIL4R, CDH1, MIR214, NEDD4L, TF, BRD2, BRD3, BRDT, DECR1, GLRX5, NCOA3, NR5A2, PANX2, RHEBP1, TFAP2A, CP, ARF6, GDF15, ABHD12, PPP1R13L, TFAM, KDM3B, RNF113A , AHCY, circ-TTBK2, MIR522, IDH2, PPARA, NOS2, SIAH2, RELA, PRKAA2, VDR, NEDD4, PRDX1, AR, MTF1, COPZ1, NUPR1, USP35, NEAT1, PARP1, PARP2, PARP3, PARP4, PARP6, PARP8, PARP9, PARP10, PARP11, PARP12, PARP14, PARP15, PARP16, PDSS2, TXN, SENP1, OIP5-AS1, MIR190A, FGF21, CREB1, CREB3, CREB5, GOT1, TFRC, MIR130B, BEX1, ASAH2, FABP4, AKT1S1, MLST8, SIRT1, TYRO3, SIRT6, TMSB4X, TMSB4Y, KIF20A, ECH1, circRHOT1, ETV4, MEG8, VCP, circ_0007142, RBMS1, KDM4A, MGST1, circKIF4A, miR-7-5p, circ_0067934, MPC1, CHMP1A, CAMKK2, SOX2, SRSF9, PROK2, MIR4443, SIRT2, circRNA1615, MIR27A, MIR670, MEF2C, EZH2, PEDS1, SMPD1, ADAMTS13, CDC25A, G6PD, CircFNDC3B, PPARD, ENO3, LCN2, MARCHF5, TRIB2, DHODH, MIR545, PDK4, CircPVT1, MIR9-3HG, circDTL, mmu_circRNA_0000309, IL6, PTPN18, ABCC5, CISD3, MS4A15, FURIN, circRHBG, GALNT14, KLHDC3, LINC01833, circGFRA1, MAPKAP1, PRR5, RICTOR, GSTM1, TERT, circ0097009, TMEM161B-DT, ADIPOQ, USP11, circEPSTI1, MIR18A, RARRES2 | PTGS2, TF, CHAC1, SLC40A1, TFRC, FTH1, GPX4, HSPB1, NFE2L2 |
