# Supplementary material for: Identification and verification of ferroptosis-related core gene in postmenopausal osteoporosis based on bioinformatics analysis
Source: PeerJ. 2026 Mar 31;14:e20666. doi: 10.7717/peerj.20666 (PMC13048226; doi:10.7717/peerj.20666)
Supplement: Supplemental Information 3 [file peerj-14-20666-s003.docx]

Supplementary Table S3

The ROC curves of PTEN was plotted using the GSE56116 and GSE7429 datasets.

| Datasets | Samples | Patients | Group | PTEN |
| --- | --- | --- | --- | --- |
| GSE56116 | GSM1356159 | postmenopausal healthy | Control | 777.9166667 |
|  | GSM1356166 | postmenopausal healthy | Control | 2057.5 |
|  | GSM1356167 | postmenopausal healthy | Control | 1369 |
|  | GSM1356156 | Postmenopausal osteoporosis | PMOP | 2501.666667 |
|  | GSM1356162 | Postmenopausal osteoporosis | PMOP | 1906.75 |
|  | GSM1356165 | Postmenopausal osteoporosis | PMOP | 2055.833333 |
| Datasets | Samples | Patients | Group | PTEN |
| GSE7429 | GSM179758 | Bcell-highBMD | Control | 7.048099352 |
|  | GSM179759 | Bcell-highBMD | Control | 6.875365351 |
|  | GSM179760 | Bcell-highBMD | Control | 6.741550249 |
|  | GSM179761 | Bcell-highBMD | Control | 6.738008114 |
|  | GSM179762 | Bcell-highBMD | Control | 6.914484958 |
|  | GSM179763 | Bcell-highBMD | Control | 6.959928308 |
|  | GSM179764 | Bcell-highBMD | Control | 6.939458023 |
|  | GSM179765 | Bcell-highBMD | Control | 6.832136369 |
|  | GSM179766 | Bcell-highBMD | Control | 6.732728613 |
|  | GSM179767 | Bcell-highBMD | Control | 6.982835559 |
|  | GSM179768 | Bcell-lowBMD | PMOP | 6.960111159 |
|  | GSM179769 | Bcell-low BMD | PMOP | 6.851495617 |
|  | GSM179770 | Bcell-lowBMD | PMOP | 7.068867081 |
|  | GSM179771 | Bcell-lowBMD | PMOP | 7.008061935 |
|  | GSM179772 | Bcell-lowBMD | PMOP | 6.99838587 |
|  | GSM179773 | Bcell-lowBMD | PMOP | 6.698968673 |
|  | GSM179774 | Bcell-lowBMD | PMOP | 7.00621042 |
|  | GSM179775 | Bcell-lowBMD | PMOP | 7.147962876 |
|  | GSM179776 | Bcell-lowBMD | PMOP | 6.826566733 |
|  | GSM179777 | Bcell-lowBMD | PMOP | 7.046604094 |
