# Supplementary material for: Identification and verification of ferroptosis-related core gene in postmenopausal osteoporosis based on bioinformatics analysis
Source: PeerJ. 2026 Mar 31;14:e20666. doi: 10.7717/peerj.20666 (PMC13048226; doi:10.7717/peerj.20666)
Supplement: Supplemental Information 4 [file peerj-14-20666-s004.docx]

Supplementary Table S4

The proportion of 22 kinds of immune cell types in GSE230665 dataset.

| Cell Type | GSM7230904 | GSM7230905 | GSM7230906 | GSM7230907 | GSM7230908 | GSM7230909 | GSM7230910 | GSM7230911 | GSM7230912 | GSM7230913 | GSM7230914 | GSM7230915 | GSM7230916 | GSM7230917 | GSM7230918 |
| --- | --- | --- | --- | --- | --- | --- | --- | --- | --- | --- | --- | --- | --- | --- | --- |
| B cells naive | 0.060674323 | 0.037989461 | 0.080248108 | 0.126358176 | 0.138512533 | 0.088645318 | 0.141161308 | 0.148764709 | 0.122220201 | 0.010633421 | 0.009469345 | 0.011066811 | 0.060924111 | 0.068598295 | 0.077919073 |
| B cells memory | 0 | 0 | 0 | 0 | 0 | 0 | 0 | 0 | 0 | 0.006755617 | 0.001540302 | 0 | 0.016596065 | 0 | 0 |
| Plasma cells | 0.120074837 | 0.157485733 | 0.127357413 | 0.034822602 | 0.057038291 | 0.043332185 | 0.058873555 | 0.035474684 | 0.067751653 | 0.023732438 | 0.023733436 | 0.021574847 | 0.059730093 | 0.074485491 | 0.064130321 |
| T cells CD8 | 0 | 0 | 0 | 0 | 0.027935345 | 0 | 0.016703357 | 0.036491662 | 0 | 0 | 0 | 0 | 0 | 0 | 0 |
| T cells CD4 naive | 0.071835151 | 0.097198046 | 0.054180509 | 0.026141348 | 0 | 0.008560135 | 0.038362383 | 0.030080025 | 0.070930804 | 0.006138865 | 0 | 0.022861549 | 0.007703148 | 0.018490723 | 0.023054454 |
| T cells CD4 memory resting | 0 | 0 | 0 | 0 | 0.046696373 | 0.06800434 | 0 | 0 | 0 | 0.059923877 | 0.04400722 | 0.010969525 | 0 | 0 | 0 |
| T cells CD4 memory activated | 0.053183134 | 0.037062994 | 0.054713959 | 0.097019188 | 0.057505522 | 0.03448503 | 0.025162126 | 0.094712186 | 0.020393859 | 0 | 0 | 0 | 0.046103186 | 0.037123706 | 0.029934638 |
| T cells follicular helper | 0.016866036 | 0.015544581 | 0.009754441 | 0 | 0 | 0.003943062 | 0.018307899 | 0 | 0.022833864 | 0.014323487 | 0.014698982 | 0.015598816 | 0.029173584 | 0.022379162 | 0.021474804 |
| T cells regulatory(Tregs) | 0 | 0 | 0 | 0 | 0.01003067 | 0.017875131 | 0.001321357 | 0 | 0 | 0.021285366 | 0.023138109 | 0.021882341 | 0.01130451 | 0.011914803 | 0.014304488 |
| T cells gamma delta | 0.209395443 | 0.206847045 | 0.158981999 | 0.133851318 | 0.110761088 | 0.157514993 | 0.121578761 | 0.079705505 | 0.11344732 | 0.042254262 | 0.049341555 | 0.064002905 | 0.193605053 | 0.217886904 | 0.219658124 |
| NK cells resting | 0 | 0 | 0 | 0 | 0 | 0 | 0 | 0 | 0 | 0.033104915 | 0.025672366 | 0.029223268 | 0 | 0 | 0 |
| NK cells activated | 0 | 0 | 0 | 0 | 0.014301831 | 0.025994088 | 0.005077216 | 0 | 0.005429111 | 0 | 0 | 0 | 0.008732477 | 0.004963762 | 0 |
| Monocytes | 0.11690152 | 0.210008943 | 0.143264727 | 0.198043787 | 0.19254253 | 0.186869765 | 0.316164278 | 0.347512162 | 0.290178922 | 0 | 0 | 0 | 0 | 0 | 0 |
| Macrophages M0 | 0.144079789 | 0.108363199 | 0.110437706 | 0.205463717 | 0.14833201 | 0.210736114 | 0.13938399 | 0.096781981 | 0.150199701 | 0.672601591 | 0.708689207 | 0.69914571 | 0.214645988 | 0.214747894 | 0.242349874 |
| Macrophages M1 | 0 | 0 | 0.007709142 | 0.01345807 | 0.009644124 | 0 | 0 | 0.005180994 | 0 | 0 | 0 | 0 | 0.024567113 | 0.017062681 | 0.030549001 |
| Macrophages M2 | 0.155235163 | 0.098218219 | 0.153848553 | 0.118028531 | 0.145276921 | 0.125869602 | 0.110111541 | 0.063669427 | 0.118748772 | 0.069900302 | 0.070129681 | 0.069428192 | 0.239641702 | 0.270975944 | 0.233465943 |
| Dendritic cells resting | 0 | 0 | 0 | 0 | 0 | 0 | 0 | 0 | 0 | 0 | 0 | 0 | 0.006137115 | 0 | 0 |
| Dendritic cells activated | 0.003373576 | 0.008803274 | 0 | 0 | 0 | 0 | 0 | 0 | 0 | 0 | 0 | 0 | 0 | 0 | 0 |
| Mast cells resting | 0.030490411 | 0.017973635 | 0.074998259 | 0.046813263 | 0.041422763 | 0.028170236 | 0.006584437 | 0.061626665 | 0.017686774 | 0 | 0 | 0 | 0.081135856 | 0.041370635 | 0.04315928 |
| Mast cells activated | 0 | 0.004504868 | 0.001068526 | 0 | 0 | 0 | 0 | 0 | 0 | 0.033114689 | 0.008489542 | 0.034246035 | 0 | 0 | 0 |
| Eosinophils | 0 | 0 | 0 | 0 | 0 | 0 | 0.00120779 | 0 | 0.00017902 | 0 | 0 | 0 | 0 | 0 | 0 |
| Neutrophils | 0.017890617 | 0 | 0.023436659 | 0 | 0 | 0 | 0 | 0 | 0 | 0.00623117 | 0.021090254 | 0 | 0 | 0 | 0 |
