# Supplementary material for: Identification and verification of ferroptosis-related core gene in postmenopausal osteoporosis based on bioinformatics analysis
Source: PeerJ. 2026 Mar 31;14:e20666. doi: 10.7717/peerj.20666 (PMC13048226; doi:10.7717/peerj.20666)
Supplement: Supplemental Information 6 [file peerj-14-20666-s006.docx]

# Aipathwell immunohistochemical analysis report

| Sample | Group | Gene | Positive Cells(%) |
| --- | --- | --- | --- |
| 1 | Control | PTEN | 16.28% |
|  | Control | PTEN | 12.43% |
|  | Control | PTEN | 17.26% |
| 2 | Control | PTEN | 42.17% |
|  | Control | PTEN | 30.74% |
|  | Control | PTEN | 30.41% |
| 3 | Control | PTEN | 41.32% |
|  | Control | PTEN | 43.28% |
|  | Control | PTEN | 46.89% |
| 1 | OVX | PTEN | 38.26% |
|  | OVX | PTEN | 44.42% |
|  | OVX | PTEN | 43.97% |
| 2 | OVX | PTEN | 66.52% |
|  | OVX | PTEN | 76.53% |
|  | OVX | PTEN | 78.16% |
| 3 | OVX | PTEN | 77.49% |
|  | OVX | PTEN | 85.21% |
|  | OVX | PTEN | 90.73% |

# 1.Screenshots of PTEN in sample 1 of the control group


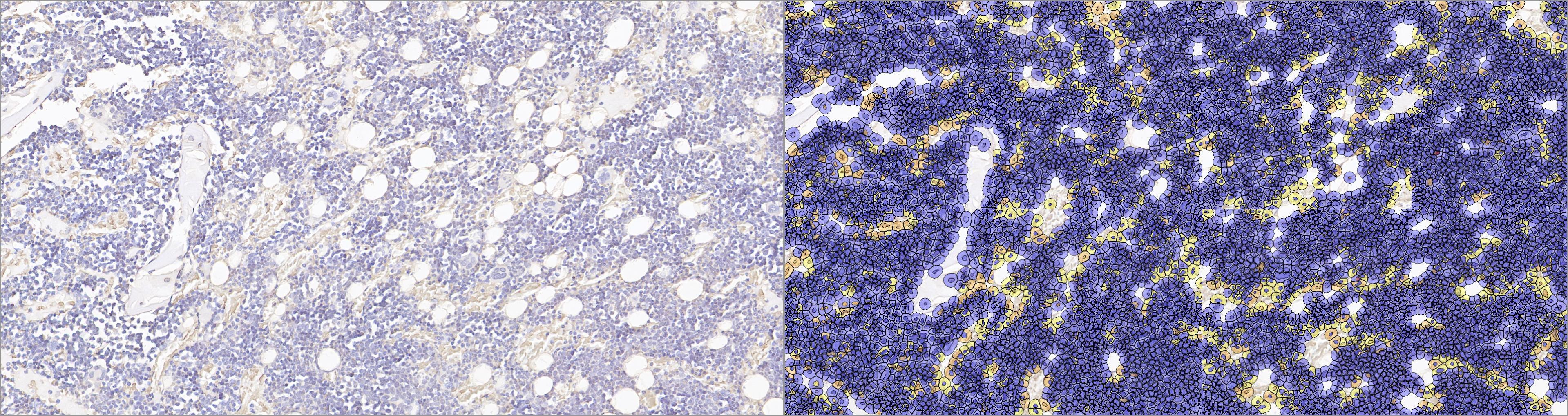


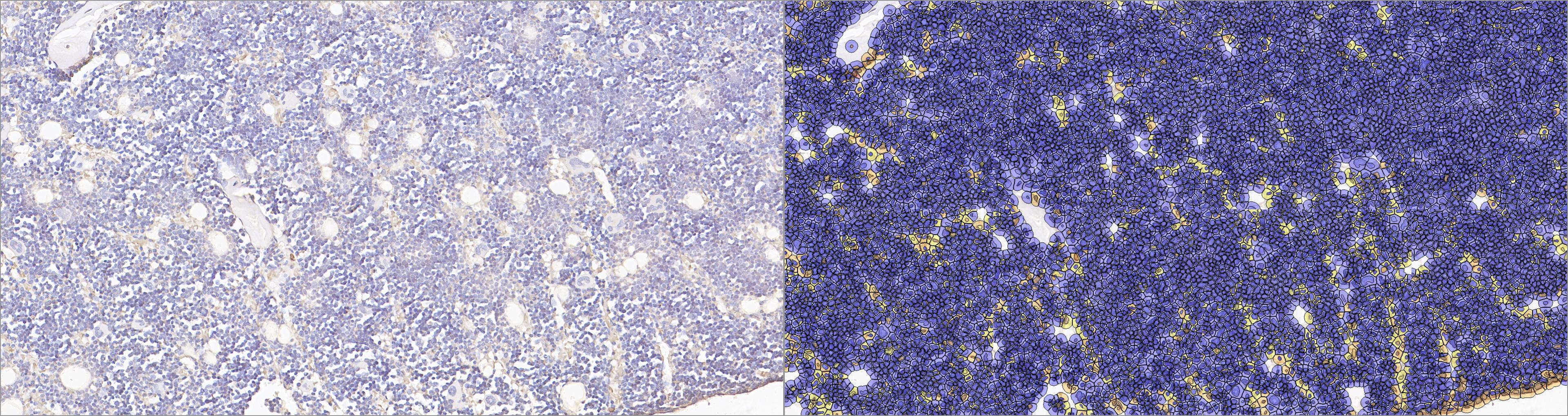


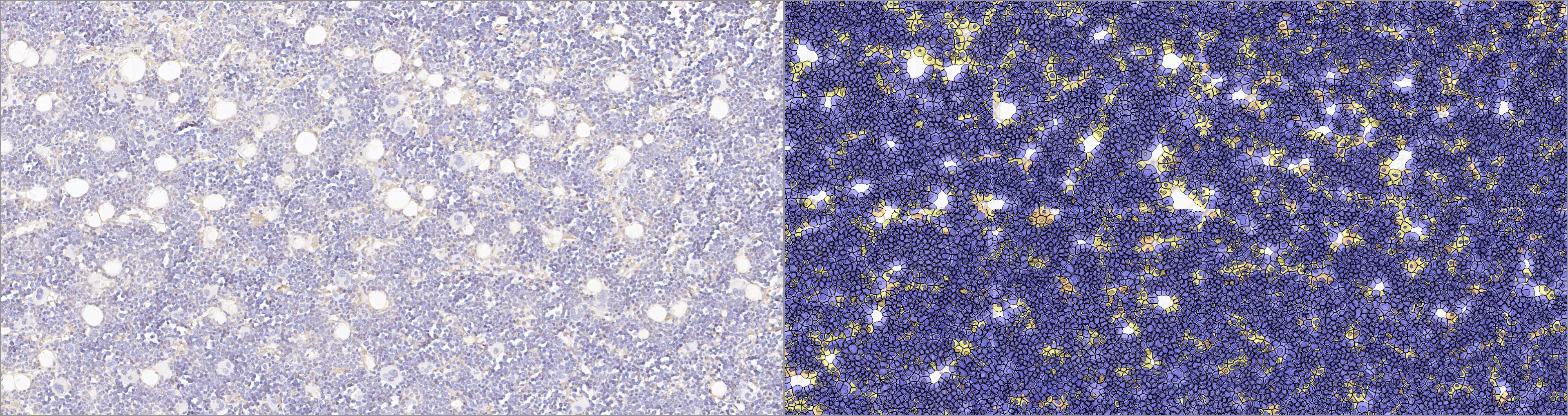


2.Screenshots of PTEN in sample 2 of the control group


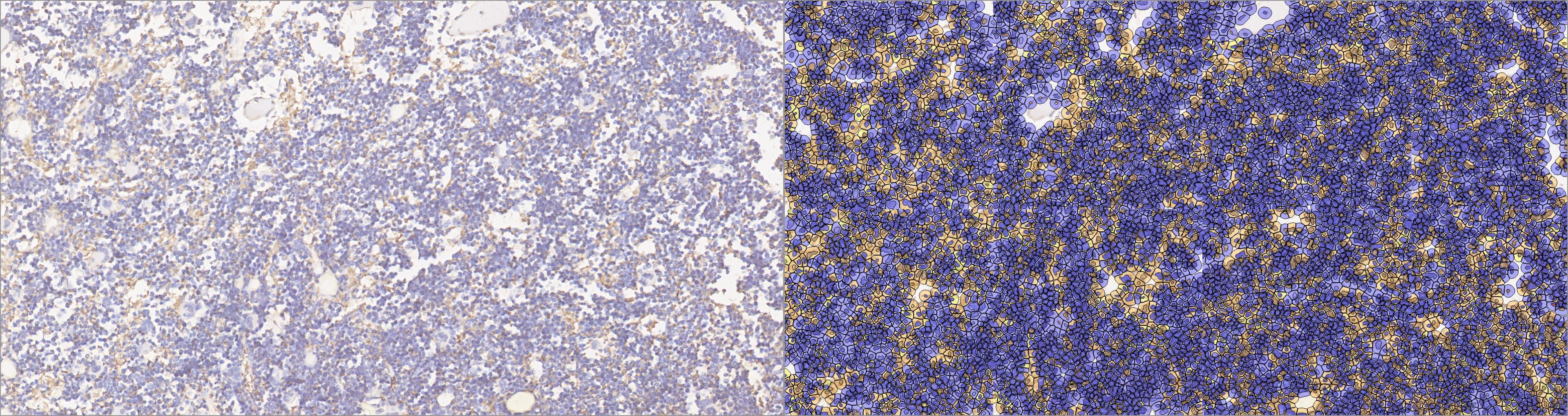


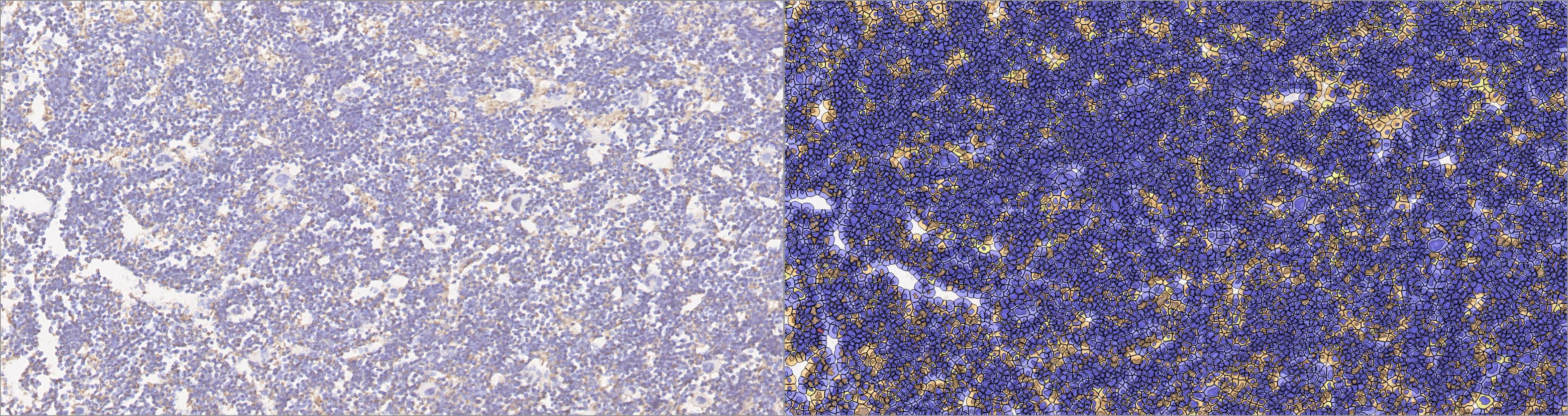


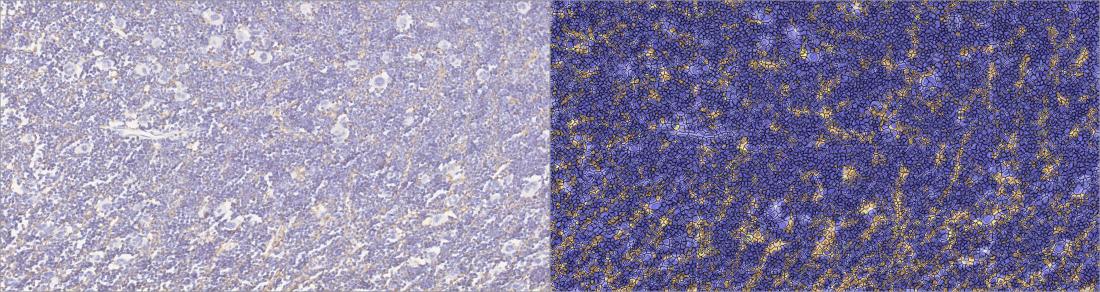


3.Screenshots of PTEN in sample 3 of the control group


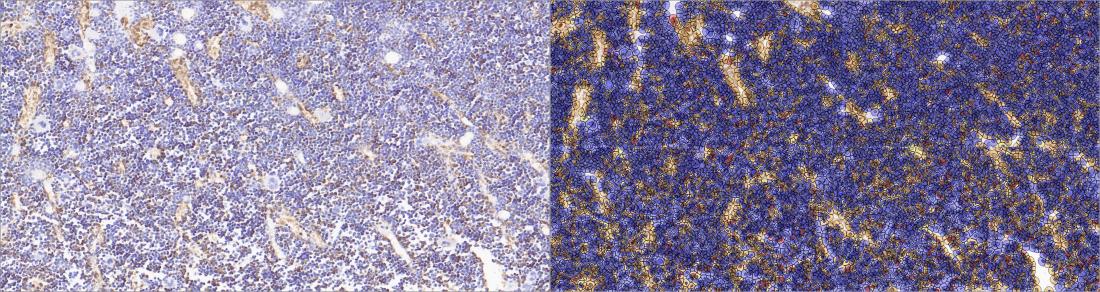


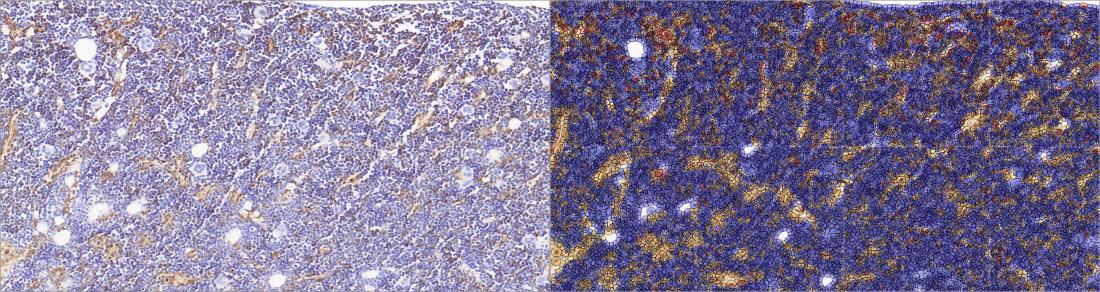


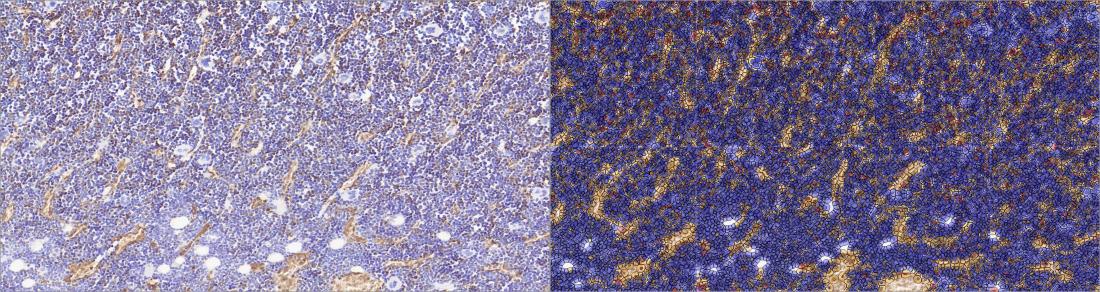


4.Screenshots of PTEN in sample 1 of the OVX group


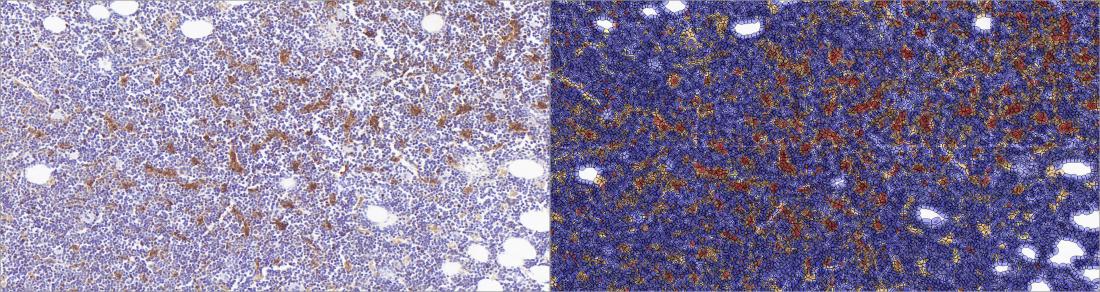


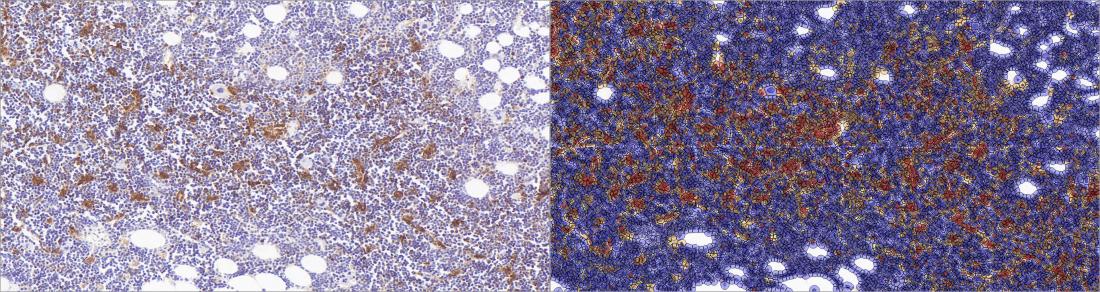


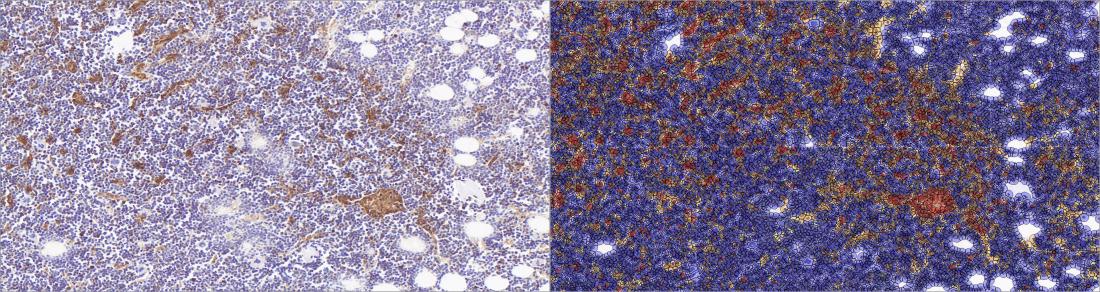


5.Screenshots of PTEN in sample 2 of the OVX group


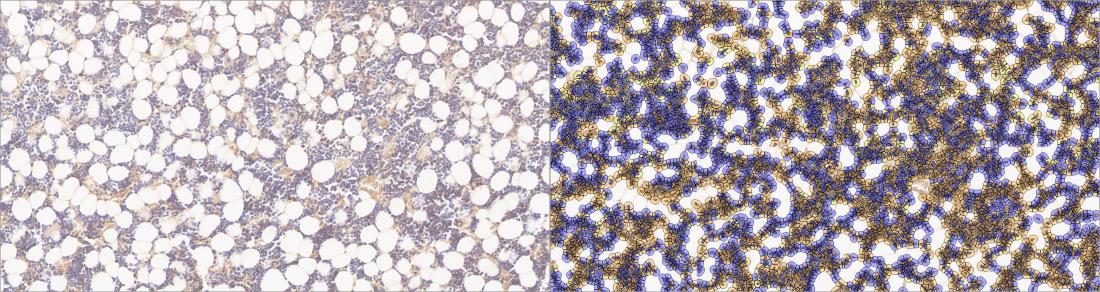


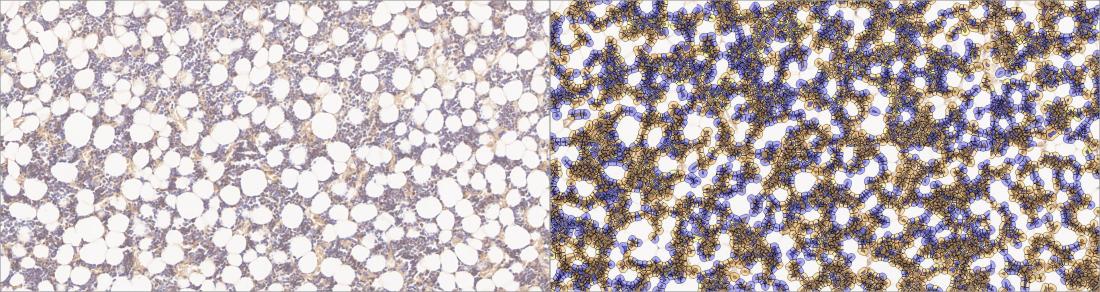


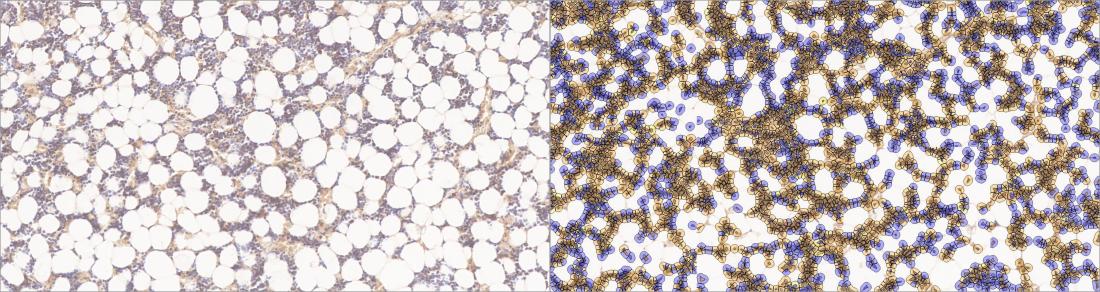


6.Screenshots of PTEN in sample 3 of the OVX group


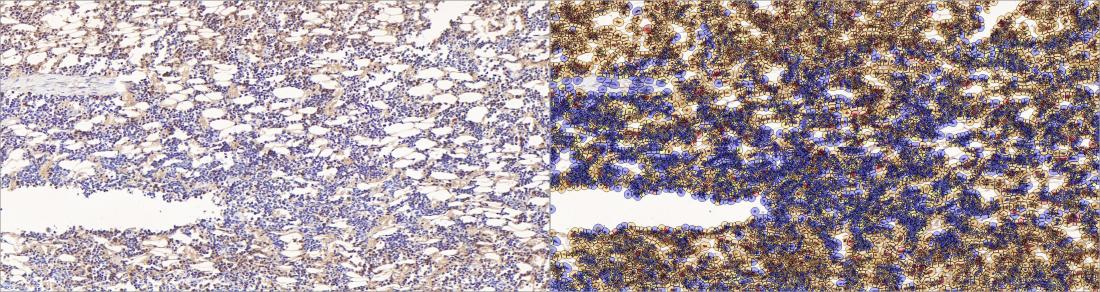


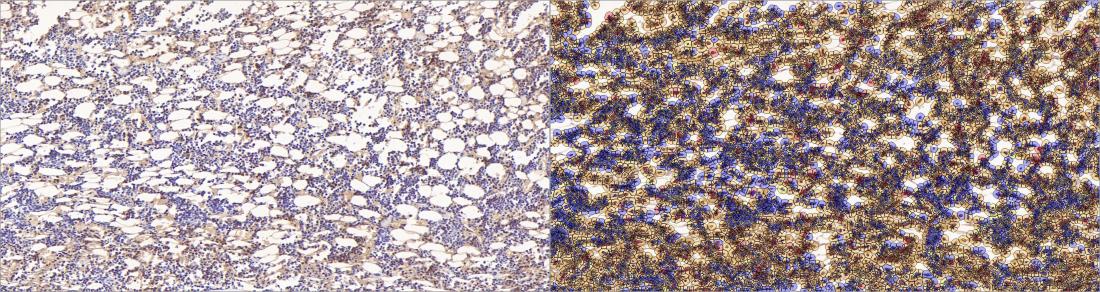


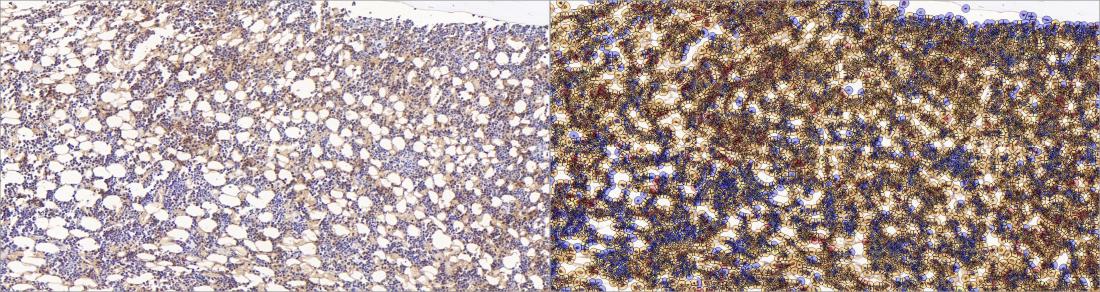


# Raw data

| Sample | Group | Positive Cell 1 Weak | Positive Cell 2 Moderate | Positive Cell 3 Strong | IOD | Total Cells Number | Positive Area, pixel | Tissue Area, pixel | Positive Intensity |
| --- | --- | --- | --- | --- | --- | --- | --- | --- | --- |
| 1 | Control | 857 | 470 | 3 | 18563 | 8169 | 56768 | 1967178 | 1 |
|  | Control | 738 | 482 | 1 | 14664 | 9824 | 40567 | 1967142 | 1 |
|  | Control | 1289 | 431 | 0 | 16188 | 9965 | 51993 | 1967106 | 1 |
| 2 | Control | 442 | 2992 | 1 | 67773 | 8146 | 155986 | 1967371 | 2 |
|  | Control | 530 | 2202 | 2 | 46351 | 8894 | 107069 | 1967395 | 2 |
|  | Control | 458 | 2441 | 1 | 46981 | 9535 | 106268 | 1967403 | 2 |
| 3 | Control | 384 | 3276 | 505 | 109646 | 10080 | 192112 | 1967327 | 2 |
|  | Control | 301 | 3505 | 778 | 148560 | 10592 | 238013 | 1967262 | 2 |
|  | Control | 362 | 3979 | 809 | 145414 | 10984 | 238666 | 1967371 | 2 |
| 1 | OVX | 604 | 2766 | 995 | 152525 | 11408 | 221119 | 1967134 | 2 |
|  | OVX | 410 | 3081 | 1349 | 187287 | 10897 | 260031 | 1967050 | 2 |
|  | OVX | 610 | 3050 | 1215 | 173498 | 11087 | 251143 | 1967242 | 2 |
| 2 | OVX | 630 | 3335 | 0 | 126092 | 5961 | 278407 | 1967034 | 2 |
|  | OVX | 280 | 3874 | 0 | 172428 | 5428 | 337778 | 1967098 | 2 |
|  | OVX | 124 | 3119 | 0 | 171981 | 4149 | 327226 | 1966670 | 2 |
| 3 | OVX | 639 | 3851 | 1282 | 217845 | 7449 | 432019 | 1966990 | 2 |
|  | OVX | 396 | 4437 | 1525 | 264364 | 7462 | 496586 | 1967102 | 2 |
|  | OVX | 343 | 5123 | 1623 | 351840 | 7813 | 645760 | 1966790 | 2 |
