# Supplementary figures and images for: Identification and verification of ferroptosis-related core gene in postmenopausal osteoporosis based on bioinformatics analysis
Source: PeerJ. 2026 Mar 31;14:e20666. doi: 10.7717/peerj.20666 (PMC13048226; doi:10.7717/peerj.20666)

Degree

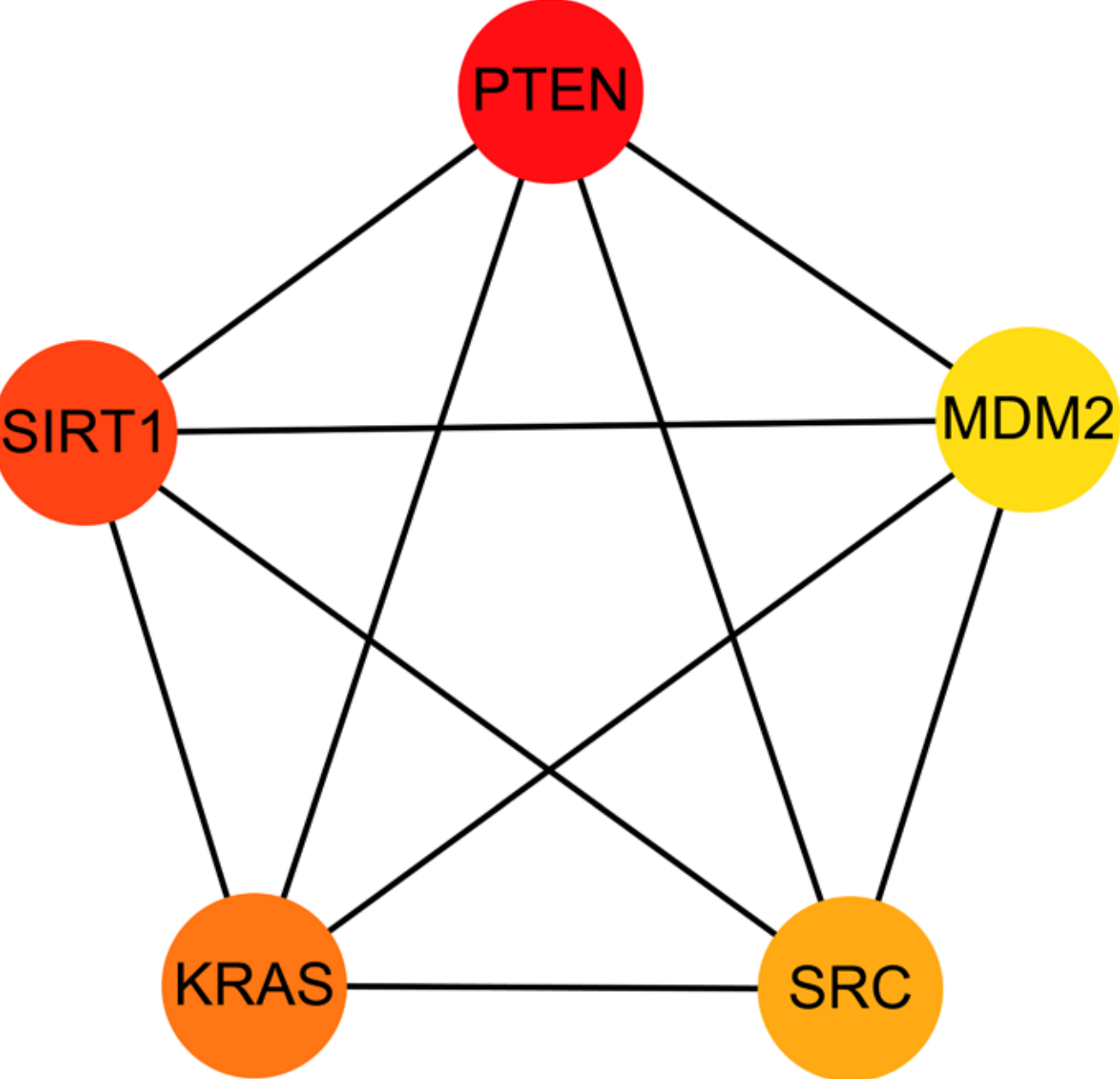

BottleNeck

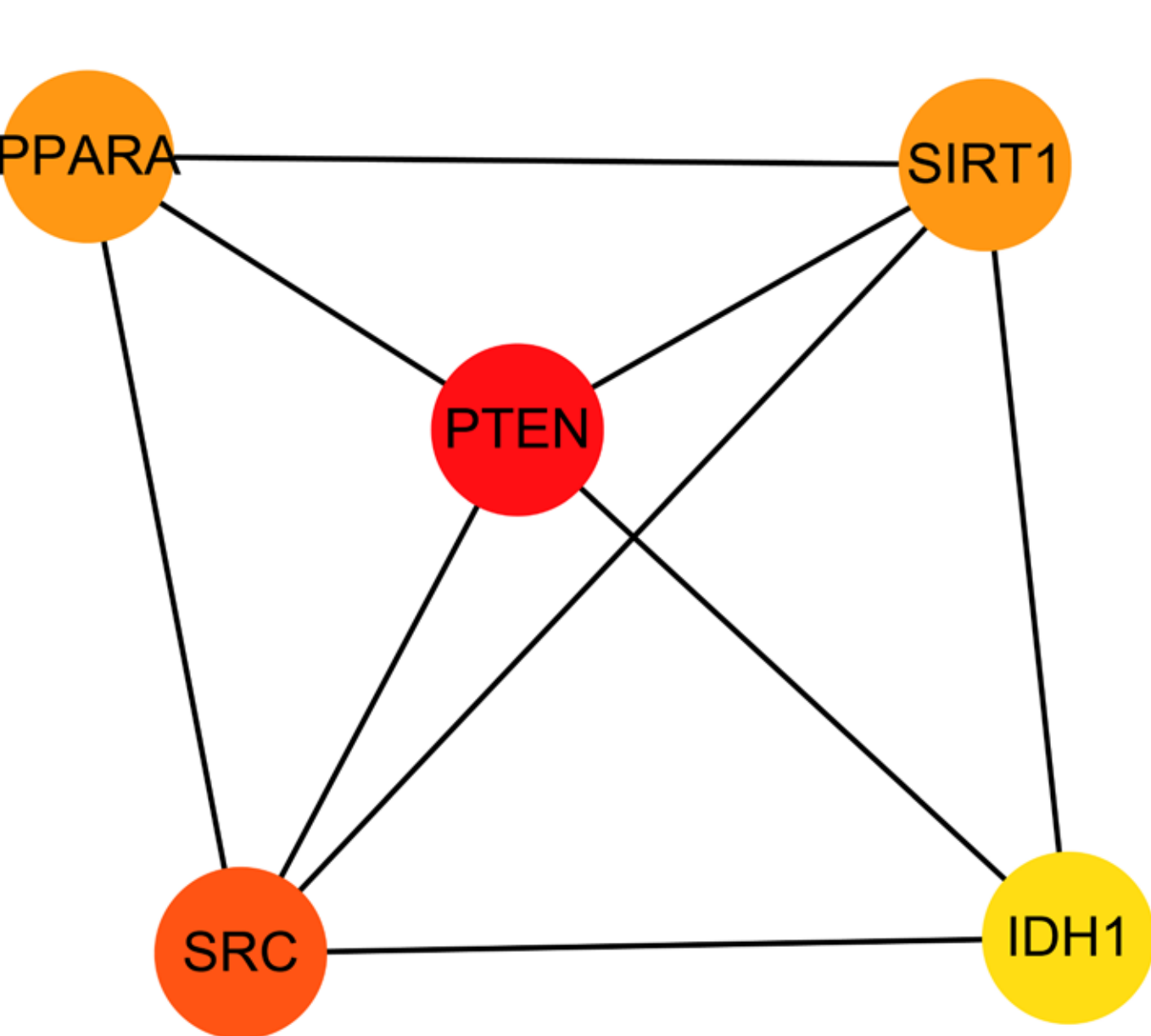

EcCentricity

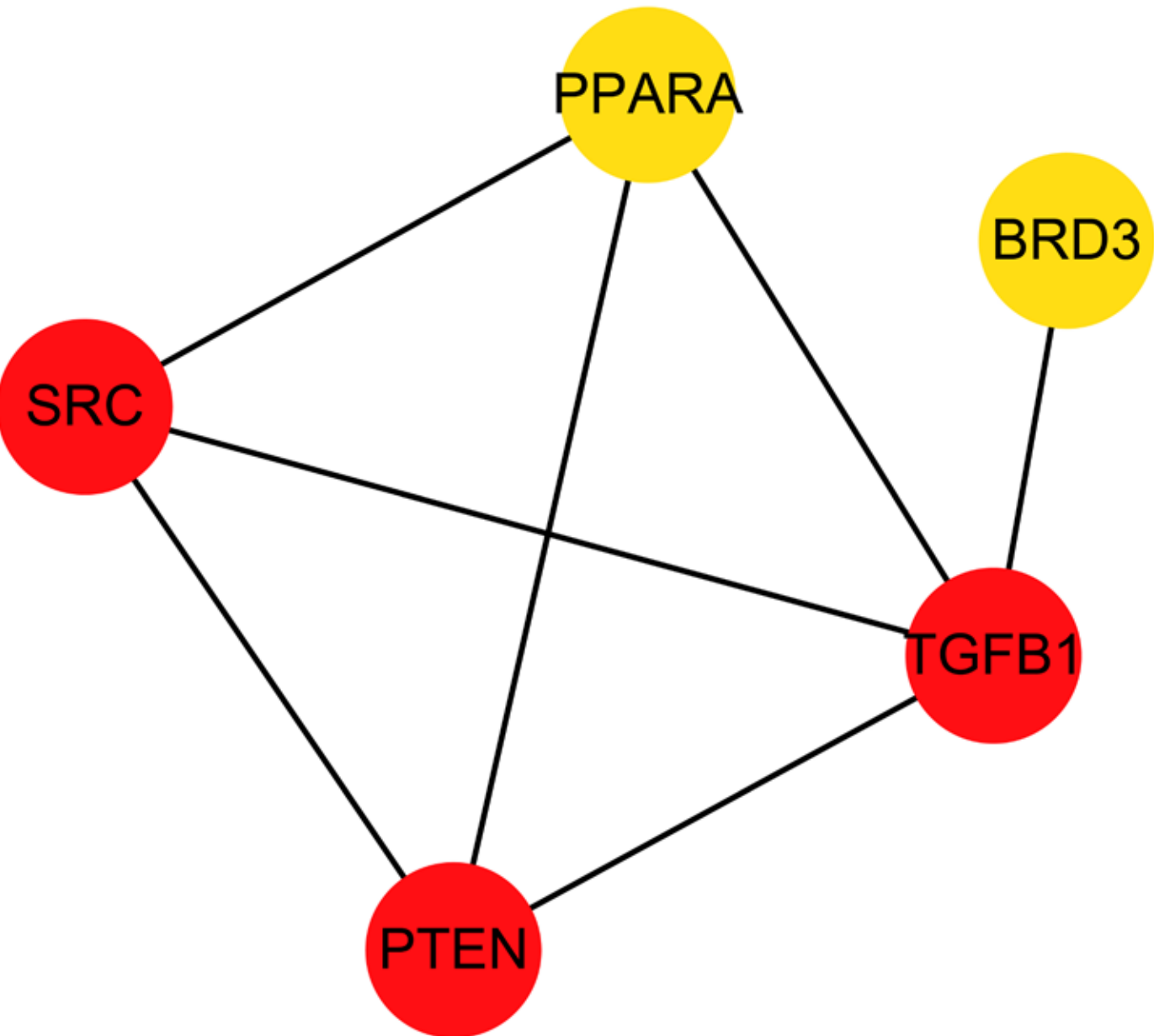

MCC

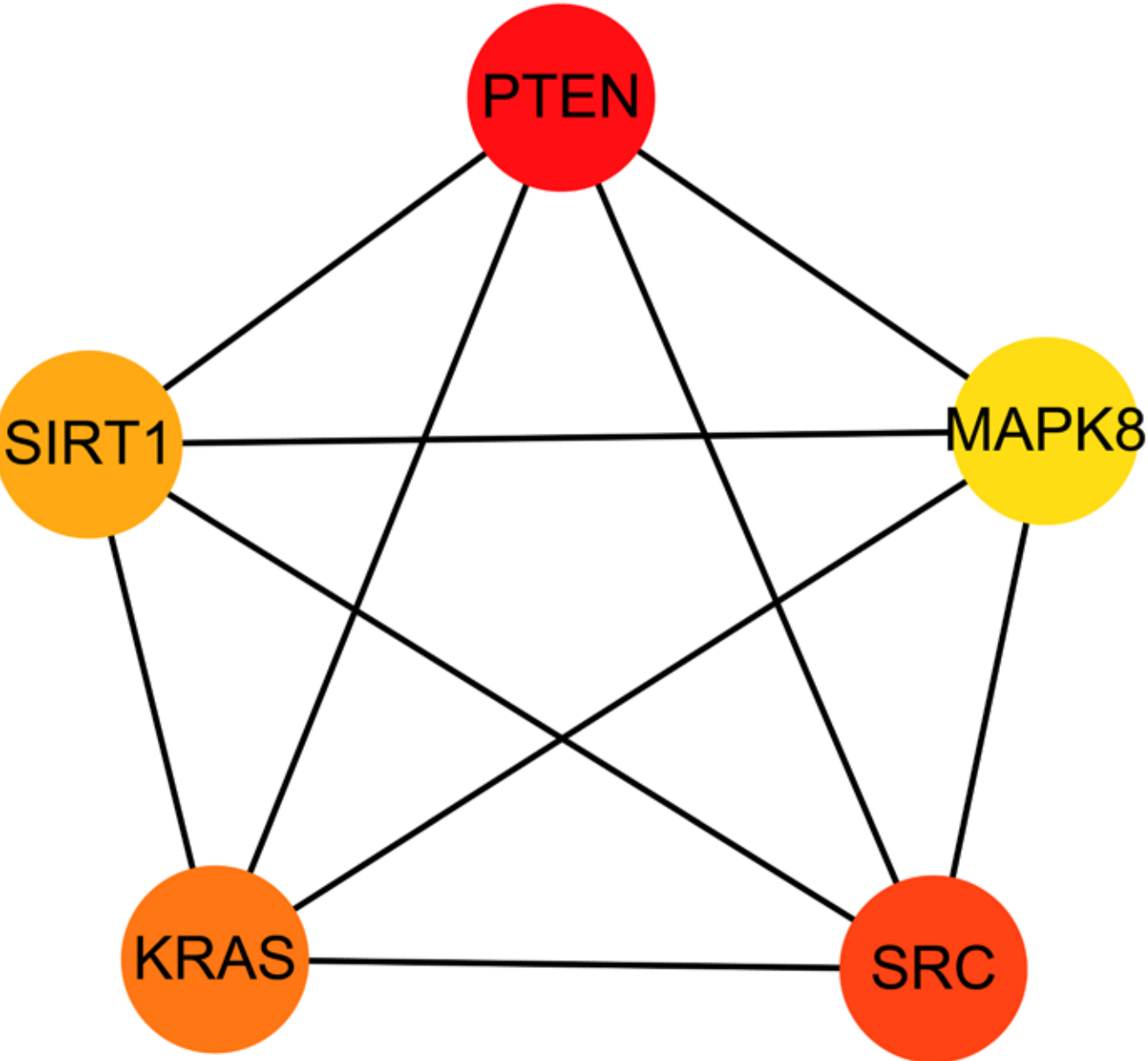

MNC

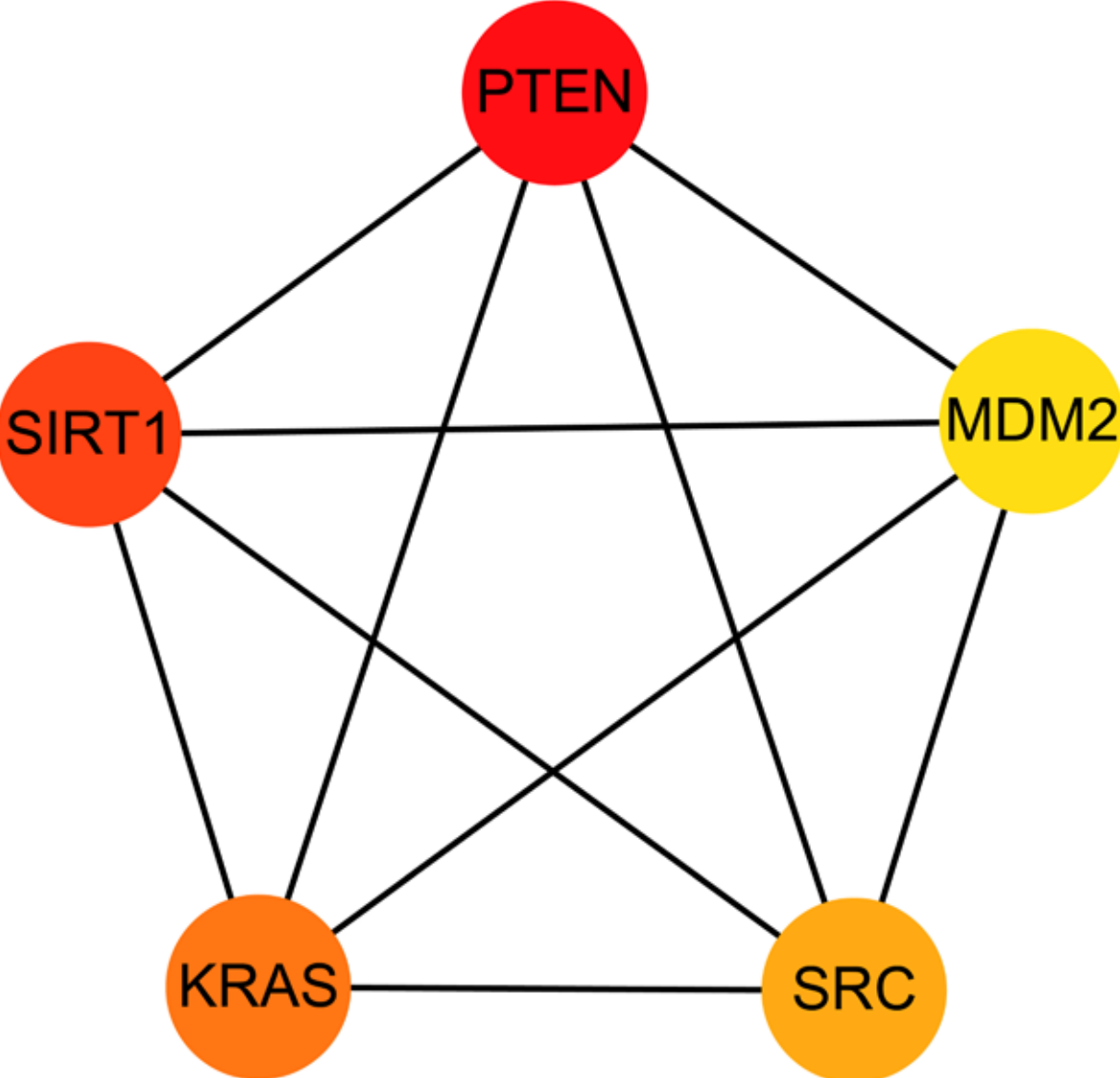

Stress

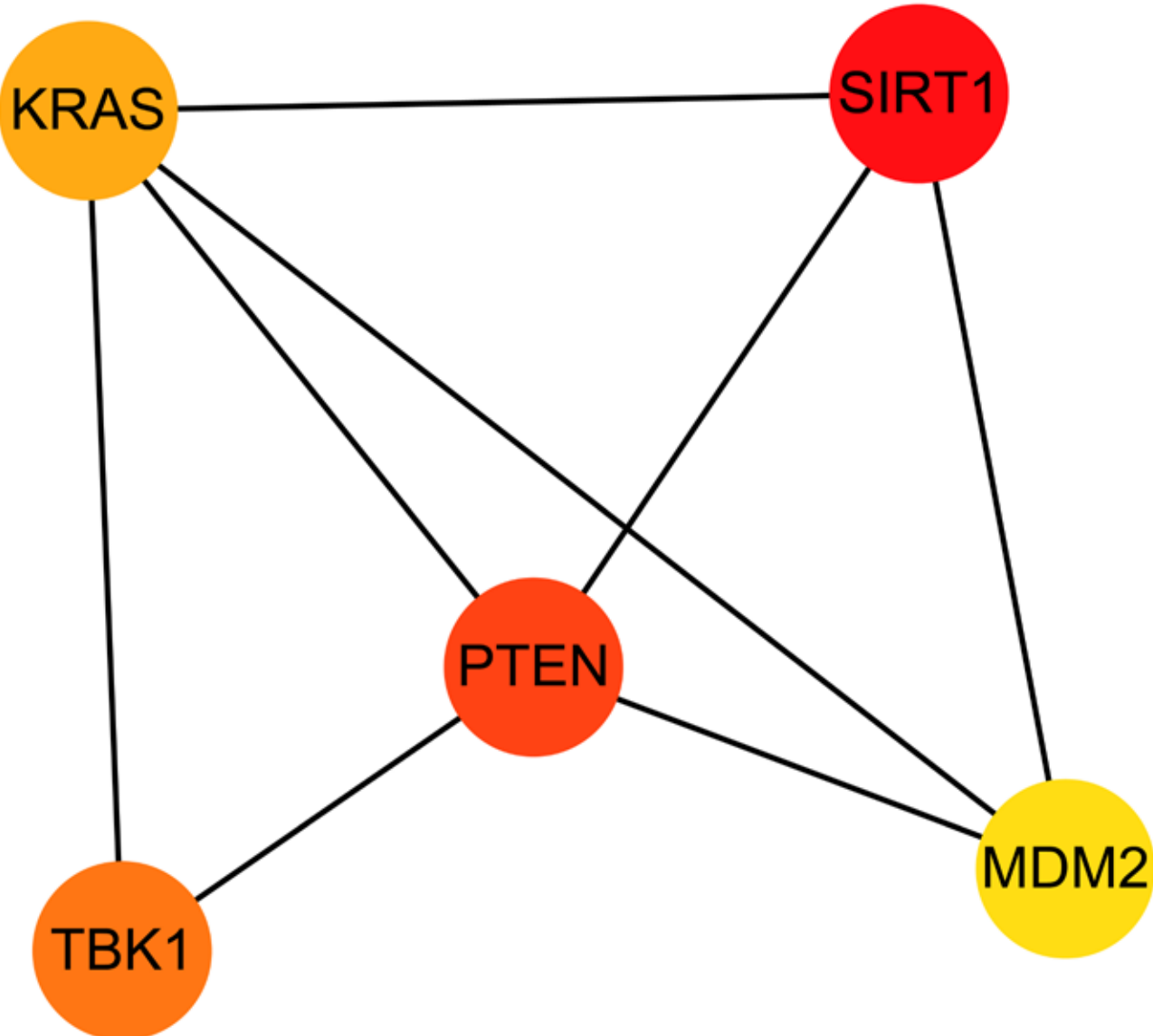

Supplement: Supplemental Information 7 [file peerj-14-20666-s007.pdf]
