# Supplementary material for: Identification and verification of ferroptosis-related core gene in postmenopausal osteoporosis based on bioinformatics analysis
Source: PeerJ. 2026 Mar 31;14:e20666. doi: 10.7717/peerj.20666 (PMC13048226; doi:10.7717/peerj.20666)
Supplement: Supplemental Information 8 [file peerj-14-20666-s008.docx]

Experimental steps of qRT-PCR

1、Total RNA extraction (Both pipette tips and centrifuge tubes are sterile and enzyme-free).

1）Take the grinding tube, add 1ml of RNA extract, add 3 3mm grinding beads, and pre-cool it on ice.

2）Take 5 to 20 mg of tissue and add it to the grinding tube.

3）Grind thoroughly with a grinder until no visible tissue blocks are left.

4）Centrifuge at 12,000 RPM for 10 minutes at 4℃ and take the supernatant.

5）Add 100 μl of chloroform substitute, invert the centrifuge tube for 15 seconds, mix thoroughly, and let it stand for 3 minutes.

6）Centrifuge at 12,000 RPM for 10 minutes at 4℃.

7）Transfer 400 μl of the supernatant to a new centrifuge tube, add 550 μl of isopropyl alcohol, and invert to mix well.

8）Place at -20℃ for 15 minutes.

9）Centrifuge at 12,000 RPM for 10 minutes at 4℃. The white precipitate at the bottom of the tube is RNA.

10）Aspirate the liquid, add 1ml of 75% ethanol, invert and mix well, then wash the precipitate.

11）Centrifuge at 12,000 RPM for 5 minutes at 4℃.

12）Repeat steps 10-11 once.

13）Aspirate the liquid completely and place the centrifuge tube on the laminar flow hood to blow for 3 to 5 minutes.

14）Dissolve the RNA in 15μl of RNA dissolving solution.

15）Detection of RNA concentration and purity using Nanodrop 2000: After zeroing the instrument blank, take 2.5μl of the RNA solution to be tested on the detection base, lower the sample arm, and start the absorbance value detection using the software on the computer.

16）Dilute the overly concentrated RNA in an appropriate proportion to achieve a final concentration of 200 ng/μl.

2、Reverse transcription (both pipette tips and PCR reaction tubes use sterile and enzyme-free types)

1）Preparation of reverse transcription reaction system (20 μL reaction system)

| Component | Volume |
| --- | --- |
| 5×SweScript All-in-One SuperMix for qPCR | 4 μL |
| gDNA Remover | 1 μL |
| Total RNA * | 10 μL |
| Nuclease-Free Water | Add to 20 μL |

2）Gently mix and centrifuge

3）The reverse transcription program was set up and reverse transcription was completed on a regular PCR instrument.

| Temperature | Time |
| --- | --- |
| 25℃ | 5 min |
| 42℃ | 30 min |
| 85℃ | 5 sec |

3、Quantitative PCR

1）Primer information:

Identification of upstream primers: -S (sense primer) or -F (forward primer);

Identification of downstream primers: -A (antisense primer) or -R (reverse primer);

| Primer information | Primer name | Primer sequence (5'-3') | Fragment length (bp) | Annealing temperature (℃) |
| --- | --- | --- | --- | --- |
| NM_017008.4 | R-GAPDH-S | CTGGAGAAACCTGCCAAGTATG | 138 | 60 |
|  | R-GAPDH-A | GGTGGAAGAATGGGAGTTGCT |  | 60 |
|  |  |  |  |  |
| NM_031606.1 | R-Pten（1）-S | CGTGCGGATAATGACAAGGA | 149 | 60 |
|  | R-Pten（1）-A | GGATTTGATGGCTCCTCTACTG |  | 60 |

2）Take 0.1ml of the PCR reaction plate and prepare the following reaction system. Prepare 3 tubes for each reverse transcription product. After spotting the samples, use PCR sealing film in conjunction with a sealing film instrument to complete the sealing film, and then centrifuge with a microplate centrifuge.

| 2×Universal Blue SYBR Green qPCR Master Mix | 7.5μl |
| --- | --- |
| 2.5μM gene primers (upstream + downstream) | 1.5μl |
| Reverse transcription product（cDNA） | 2.0μl |
| Water Nuclease-Free | 4.0μl |

3）PCR amplification was completed on a fluorescence quantitative PCR instrument.

| Stage1 | Stage2（40 cycles） | Stage3（Melting curve） |
| --- | --- | --- |
| 95℃, 30s Predegeneration | 95℃，15s Degeneration | 65℃→95℃ |
|  | 60℃，30s Annealing/Elongation | A fluorescence signal is collected once for every 0.5℃ increase in temperature |

4、Result processing

ΔΔCT method：

A=CT(Target gene, sample to be tested)- CT(Internal reference genes, sample to be tested)

B=CT(Target gene, control sample)- CT(Internal reference genes, control samples)

K=A-B

Expression multiple=2-K

5、Data results and processing

| First | Group | Gene | expression | Group | Gene | expression | 2-∆∆CT |
| --- | --- | --- | --- | --- | --- | --- | --- |
|  | Sham | GAPDH | 19.40 | Sham | PTEN | 27.98 | 1.031656658 |
|  | Sham | GAPDH | 19.35 | Sham | PTEN | 28.00 | 1.011739847 |
|  | Sham | GAPDH | 19.34 | Sham | PTEN | 28.08 | 0.958067173 |
|  | OVX | GAPDH | 19.57 | OVX | PTEN | 26.37 | 3.441463419 |
|  | OVX | GAPDH | 19.48 | OVX | PTEN | 26.50 | 3.152551166 |
|  | OVX | GAPDH | 19.44 | OVX | PTEN | 26.20 | 3.871471889 |
|  |  |  |  |  |  |  |  |
| Second | Group | Gene | expression | Group | Gene | expression | 2-∆∆CT |
|  | Sham | GAPDH | 19.17 | Sham | PTEN | 26.69 | 0.875941999 |
|  | Sham | GAPDH | 19.04 | Sham | PTEN | 26.47 | 1.013489601 |
|  | Sham | GAPDH | 18.90 | Sham | PTEN | 26.32 | 1.126432968 |
|  | OVX | GAPDH | 19.38 | OVX | PTEN | 25.26 | 2.817048031 |
|  | OVX | GAPDH | 19.22 | OVX | PTEN | 25.16 | 3.013753473 |
|  | OVX | GAPDH | 19.28 | OVX | PTEN | 25.48 | 2.409686933 |
|  |  |  |  |  |  |  |  |
| Third | Group | Gene | expression | Group | Gene | expression | 2-∆∆CT |
|  | Sham | GAPDH | 19.18 | Sham | PTEN | 26.60 | 1.005087201 |
|  | Sham | GAPDH | 19.21 | Sham | PTEN | 27.03 | 0.746973694 |
|  | Sham | GAPDH | 19.05 | Sham | PTEN | 26.19 | 1.331959285 |
|  | OVX | GAPDH | 19.28 | OVX | PTEN | 25.12 | 3.005272225 |
|  | OVX | GAPDH | 19.27 | OVX | PTEN | 24.98 | 3.331875123 |
|  | OVX | GAPDH | 19.21 | OVX | PTEN | 25.18 | 2.899170926 |

6、Experimental equipment and consumables

| Instrument name | Manufacturer | Item number |
| --- | --- | --- |
| High-speed cryogenic micro centrifuge | DragonLab | D3024R |
| Fluorescence quantitative PCR instrument | Bio-rad | CFX Connect |
| PCR instrument | Beijing Dongsheng Innovation Biotechnology Co., LTD | ETC811 |
| Clean bench | Su Jing An Tai | SW-CJ-1FD |
| Three-dimensional cryogenic grinding instrument | Servicebio | KZ-5F-3D |
| Grinding beads (zirconia) | Servicebio | G0203 |
| Vortex mixer | Servicebio | SMV-3500 |
| centrifuge | Servicebio | SMC-5000F |
| Anhydrous ethanol | Sinopharm Group Chemical Reagent Co., LTD | 10009218 |
| Water Nuclease-Free | Servicebio | G4700 |
| RNA dissolving solution | Servicebio | G3029 |
| SweScript All-in-One RT SuperMix for qPCR (One-Step gDNA Remover) | Servicebio | G3337 |
| 2×Universal Blue SYBR Green qPCR Master Mix | Servicebio | G3326 |
| RNA extract | Servicebio | G3013 |
| Chloroform substitutes | Servicebio | G3014 |
| Isopropyl alcohol | Sinopharm Group Chemical Reagent Co., LTD | 80109218 |
